# Supplementary material for: Mitochondrial Genomes in Perkinsus Decode Conserved Frameshifts in All Genes
Source: Mol Biol Evol. 2022 Sep 15;39(10):msac191. doi: 10.1093/molbev/msac191 (PMC9550989; doi:10.1093/molbev/msac191)
Supplement: msac191_Supplementary_Data [file msac191_supplementary_data.zip › Suppl Fig S14 - Alignment of rRNA fragments.pdf]

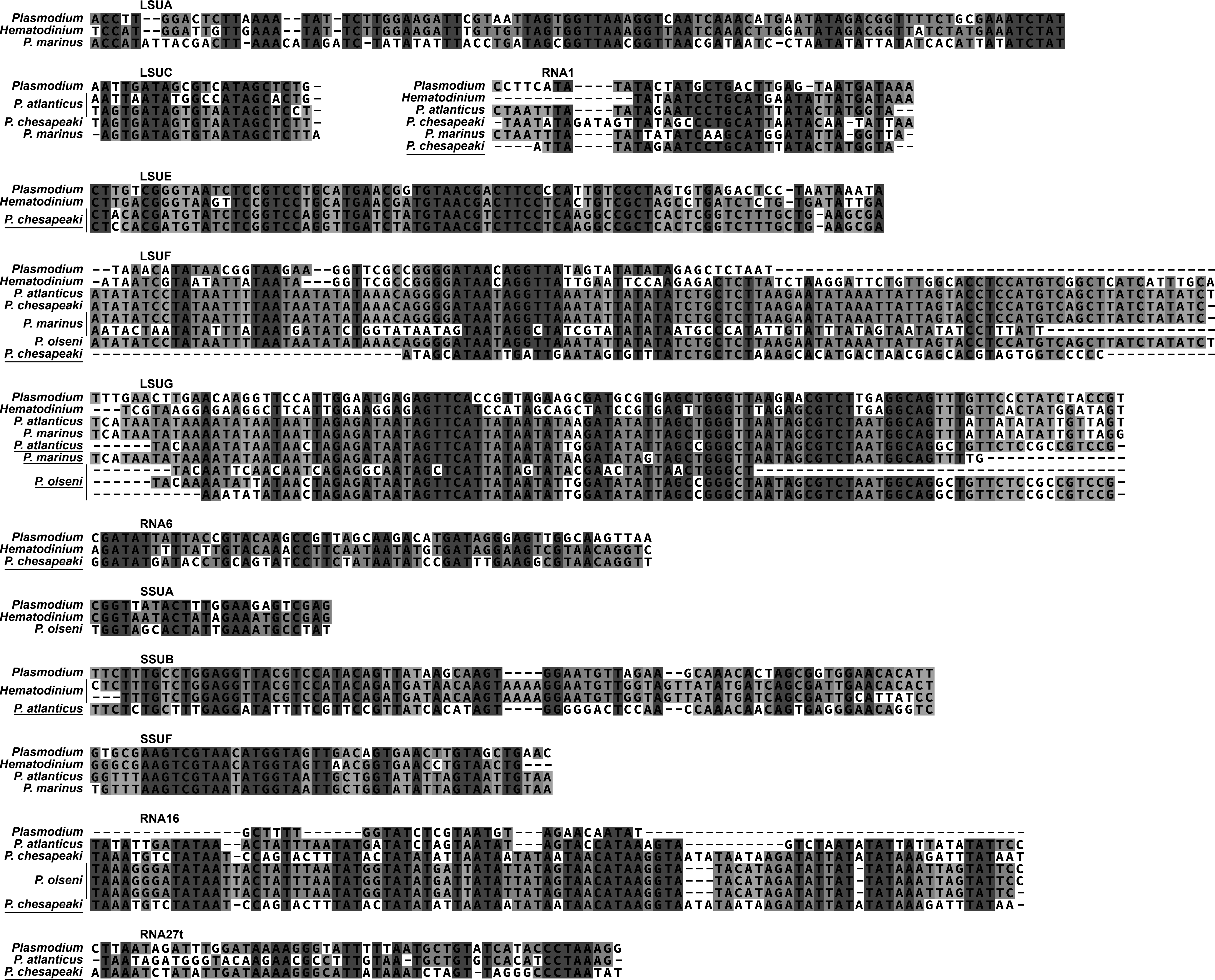
LSUA
Plasmodium
Hematodinium
P. marinus
LSUC
Plasmodium
P. atlanticus
P. chesapeaki
P. marinus
RNA1
Plasmodium
Hematodinium
P. atlanticus
P. chesapeaki
P. marinus
P. chesapeaki
LSUE
Plasmodium
Hematodinium
P. chesapeaki
LSUF
Plasmodium
Hematodinium
P. atlanticus
P. chesapeaki
P. marinus
P. olseni
P. chesapeaki
LSUG
Plasmodium
Hematodinium
P. atlanticus
P. marinus
P. atlanticus
P. marinus
P. olseni
RNA6
Plasmodium
Hematodinium
P. chesapeaki
SSUA
Plasmodium
Hematodinium
P. olseni
SSUB
Plasmodium
Hematodinium
P. atlanticus
SSUF
Plasmodium
Hematodinium
P. atlanticus
P. marinus
RNA16
Plasmodium
P. atlanticus
P. chesapeaki
P. olseni
P. chesapeaki
RNA27t
Plasmodium
P. atlanticus
P. chesapeaki
