# Supplementary material for: Mitochondrial Genomes in Perkinsus Decode Conserved Frameshifts in All Genes
Source: Mol Biol Evol. 2022 Sep 15;39(10):msac191. doi: 10.1093/molbev/msac191 (PMC9550989; doi:10.1093/molbev/msac191)
Supplement: msac191_Supplementary_Data [file msac191_supplementary_data.zip › Suppl File 3 - identification of Trp(W)-TCA_tRNAs.docx]

**Identification of alternative Trp-tRNA in *P. marinus* and *P. olseni***

Using (1) tRNAscan-SE2.0 in the nuclear genomes of *P. marinus* (PMAR) and *P. olseni* (POLS) and (2) LOTTE-Seq we identify the following Trp-tRNA like tRNAs with TCA anti-codon (TGA codon).

**(1) *tRNAscan-SE2.0:***

**genomic scaffold name tRNA begin/end type anti-codon intron begin/end Score**

PMAR_002 1389877 1389806 Sup TCA 0 0 53.3

PMAR_019 723759 723830 Sup TCA 0 0 57.0

POLS_001 413668 413597 Sup TCA 0 0 63.3

POLS_026 363056 363124 Sup TCA 0 0 37.3 (pseudo)

**(2) *LOTTE-Seq:***

No full-length LOTTE-seq reads corresponding to Trp-tRNA like tRNAs were found in read data, however, shorter reads (below the tRNAscan size threshold) were manual overlapped with loci from tRNAScan results from the genomes. Of these mapped reads secondary structures were considered and allowing identification/confirmation of low coverage alternative Trp-tRNA like tRNAs.

PMAR_002 1389861 1389814

GGTAACGCATCTAACTTCAGATCAGAAGGTTGTCCGTTCGAATCGGGC

PMAR_002 1389873 1389806

CTGTGGCGTAGTGGTAACGCATCTAACTTCAGATCAGAAGGTTGTCCGTTCGAATCGGGCCGGGGTCA

PMAR_019 723759 723830

GGCCCTGTGGCGCAACGGTAGCGCATCTGACTTCAGATCAGAAGGTTGTACGTTCGAATCGGGCCGAGGTCA

POLS_001 413665 413597

CCTGTGGCGCAACGGTAGCGCATCTGACTTCAGATCAGGAGGTTGTCCGTTCGAGTCGGGCCGGGGTCA

POLS_026 363056 363124

GGCCCTGTGGCGCAACGGTAGCGCATTTTTCAGATCAGAAGGTTGTCCGTTCGAATCGGGCCGGGGTCA

Note: in-silico predictions and LOTTE-seq data have good overlap.
