# Supplementary figures and images for: Mitochondrial Genomes in Perkinsus Decode Conserved Frameshifts in All Genes
Source: Mol Biol Evol. 2022 Sep 15;39(10):msac191. doi: 10.1093/molbev/msac191 (PMC9550989; doi:10.1093/molbev/msac191)

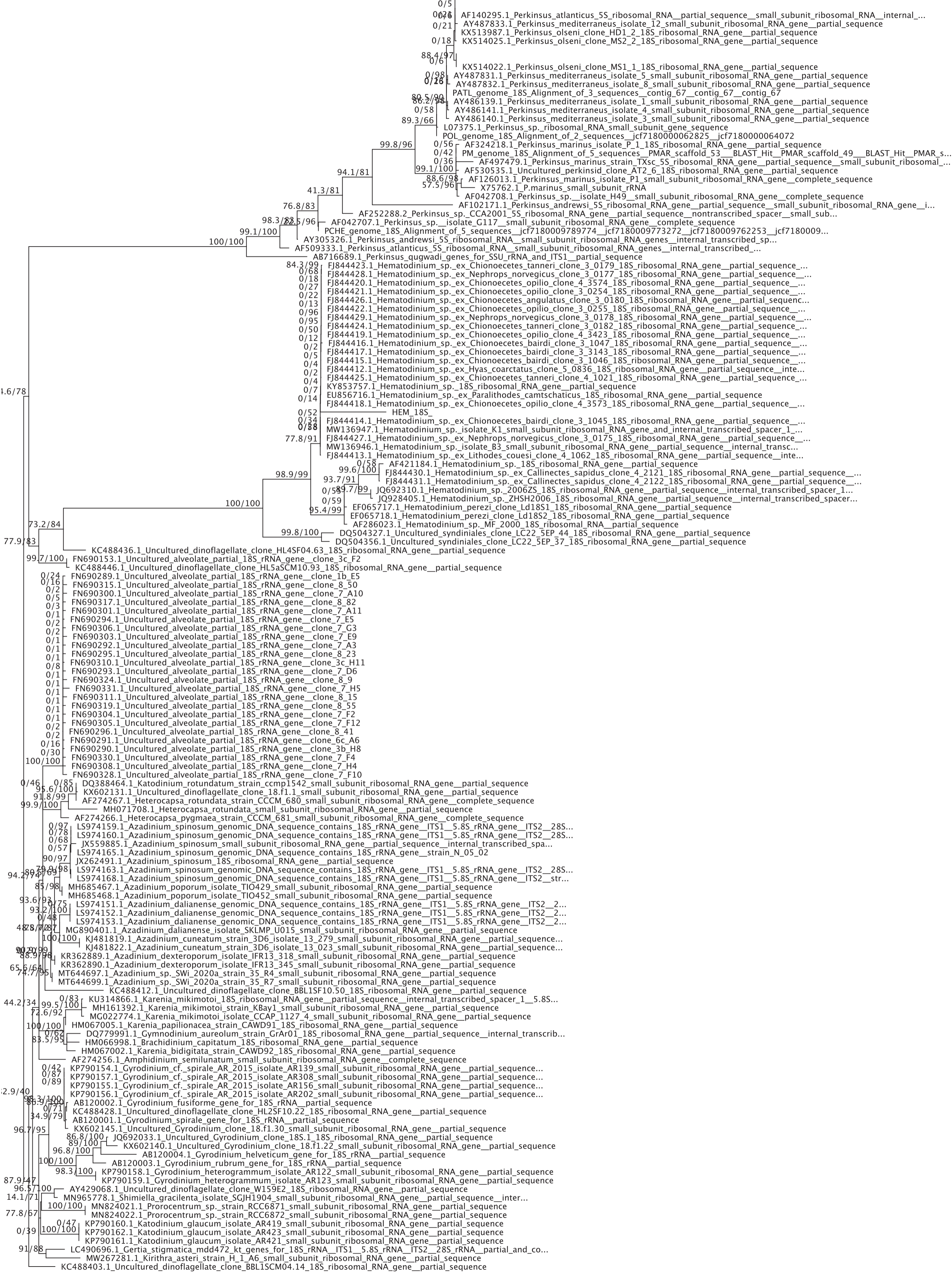

Supplement: msac191_Supplementary_Data [file msac191_supplementary_data.zip › Suppl Fig S1 - Perkinsus 18S phylogeny raw tree.pdf]

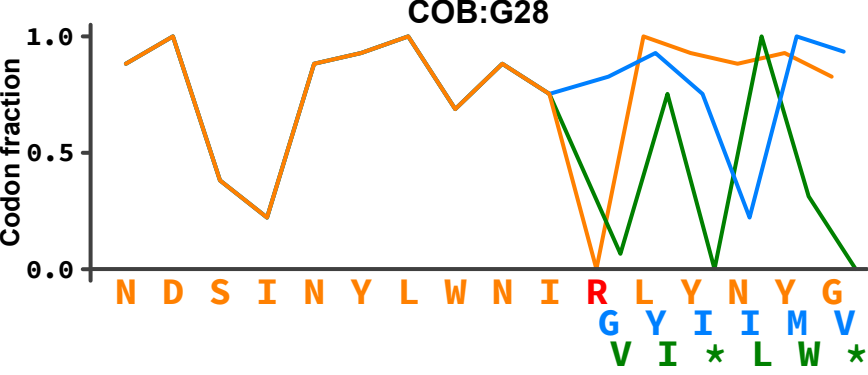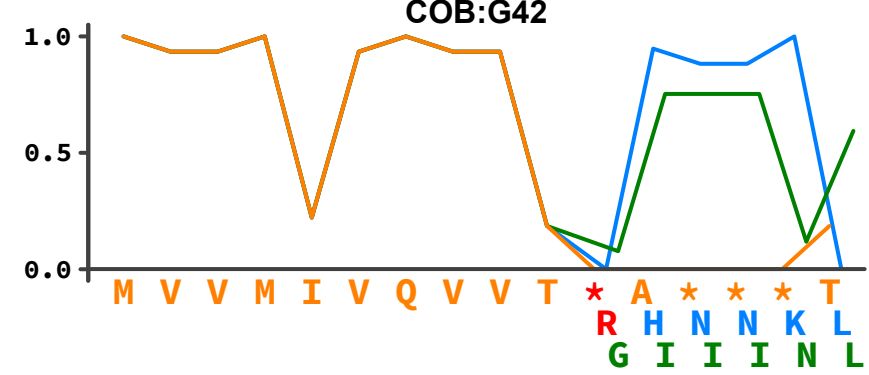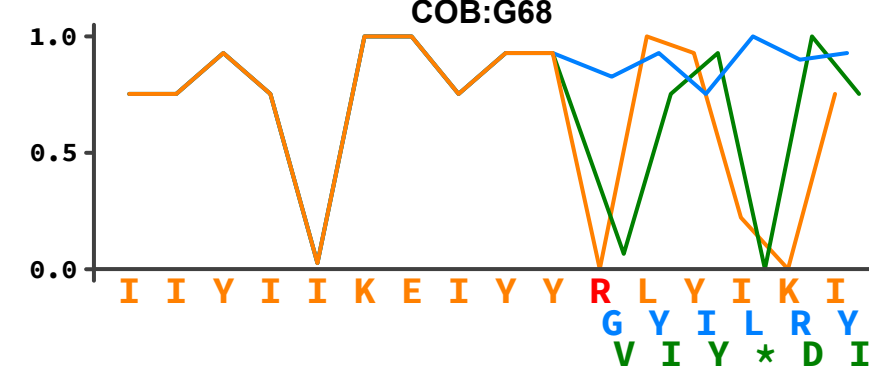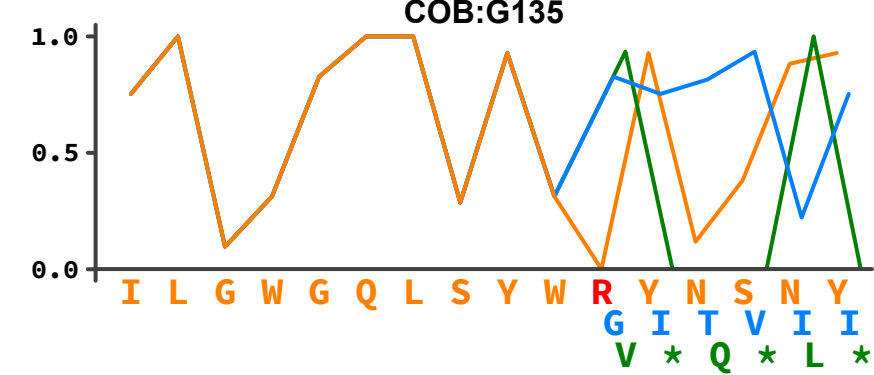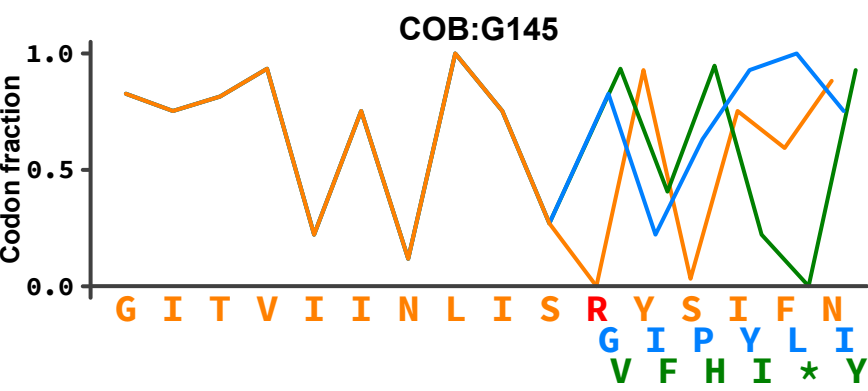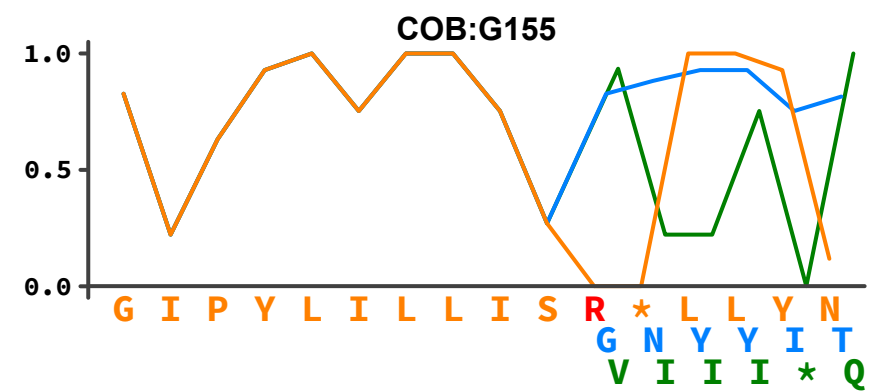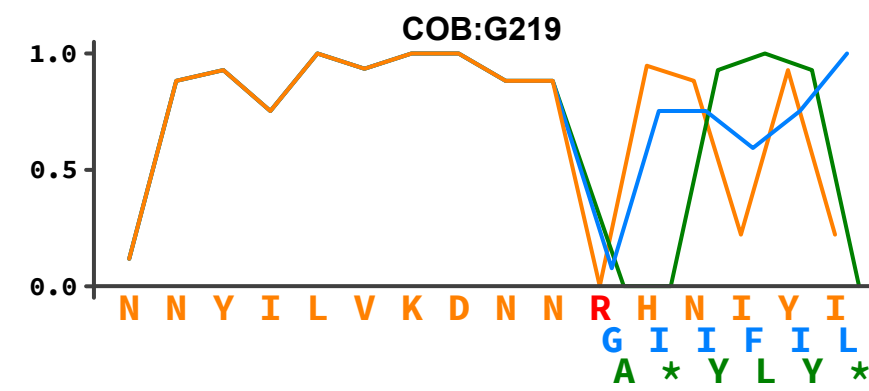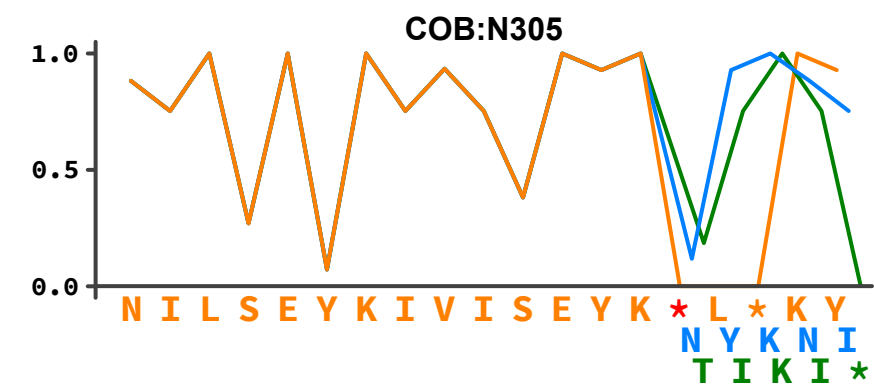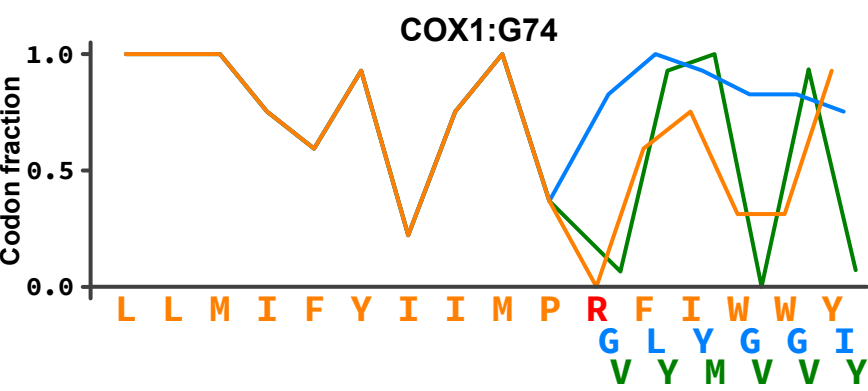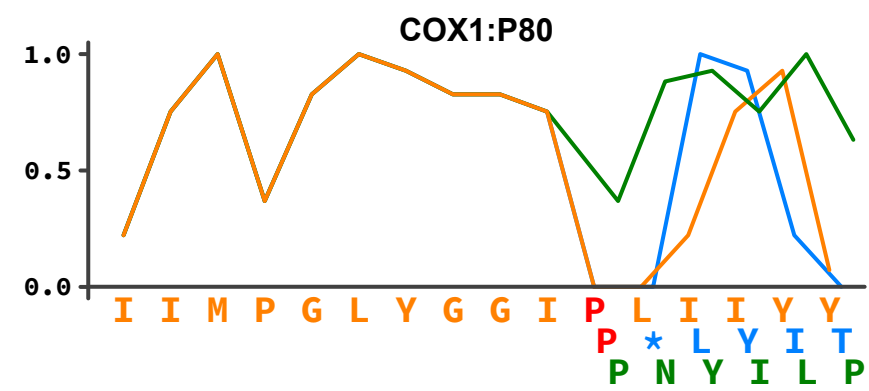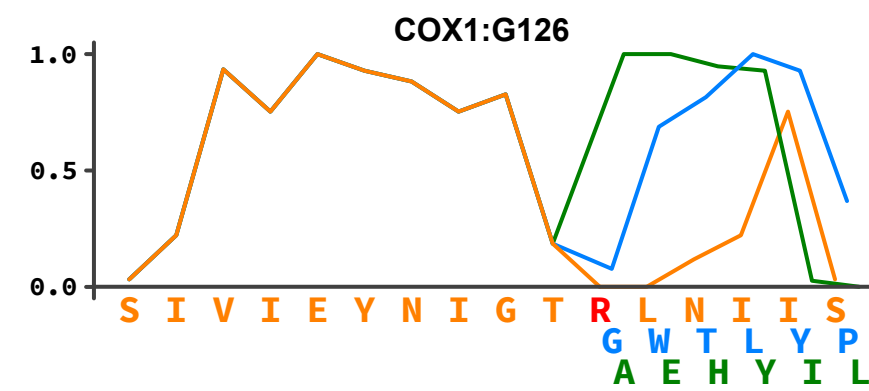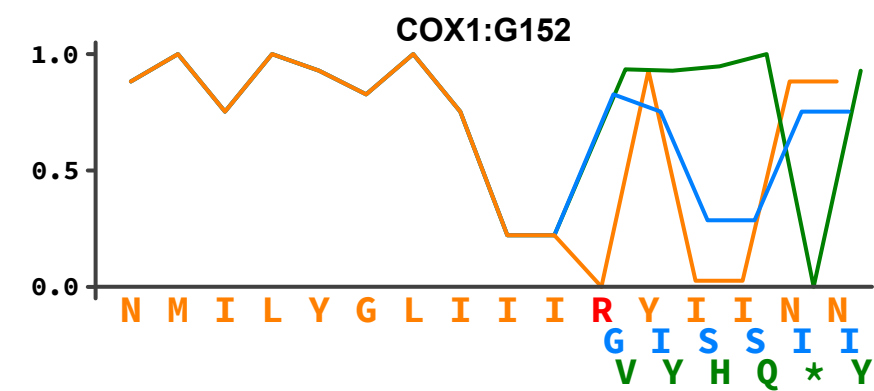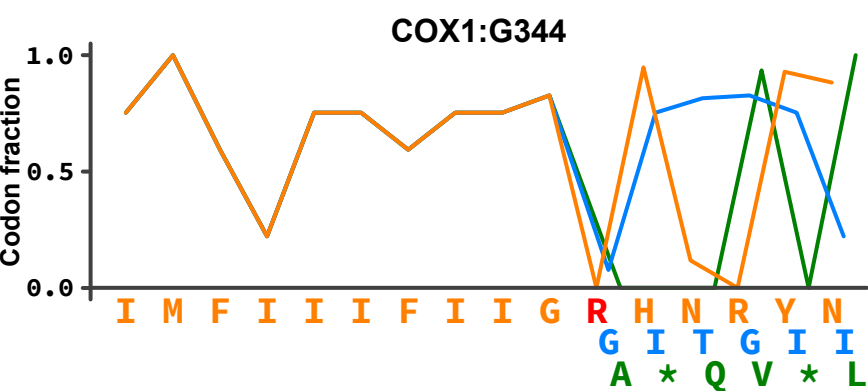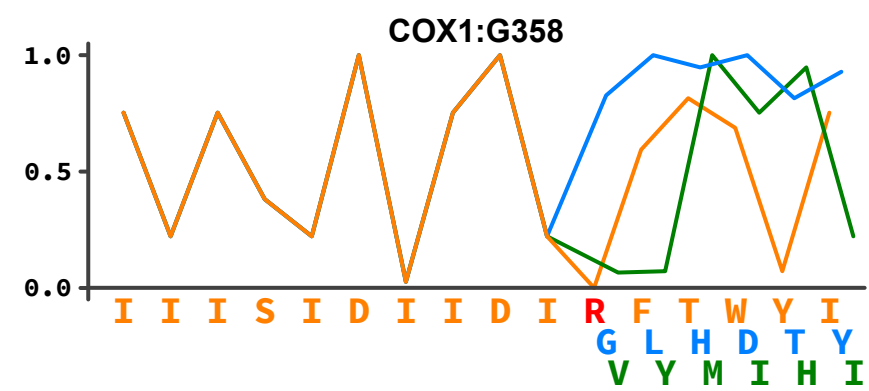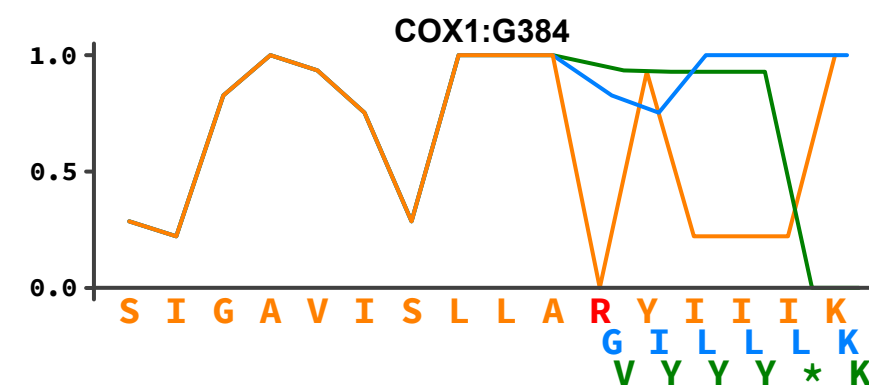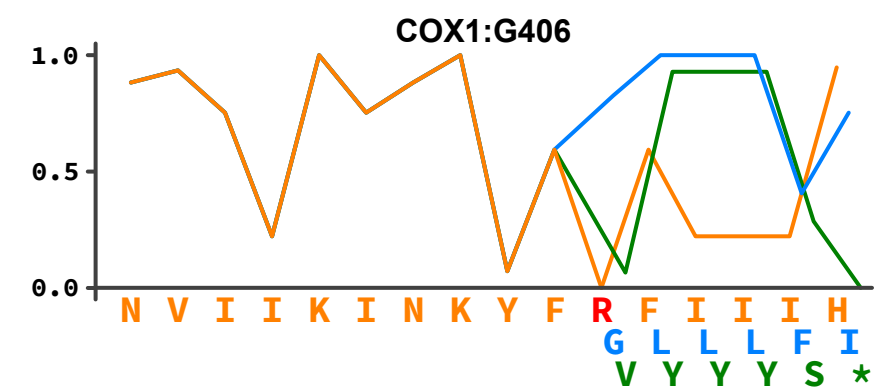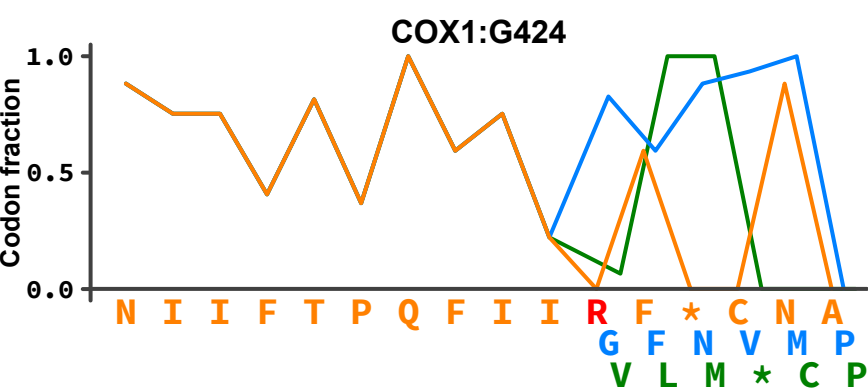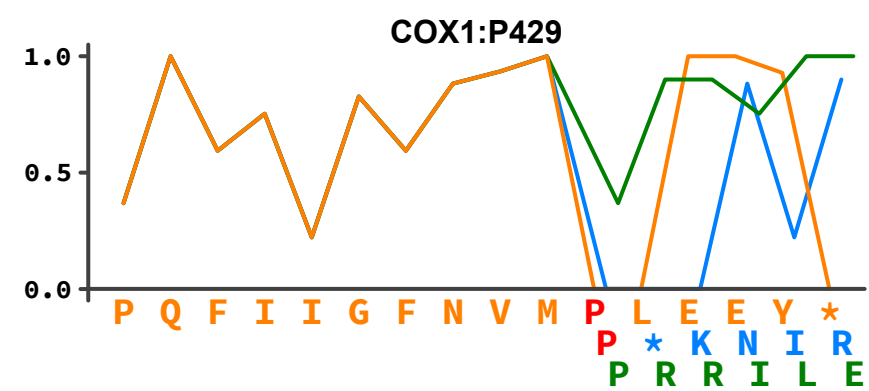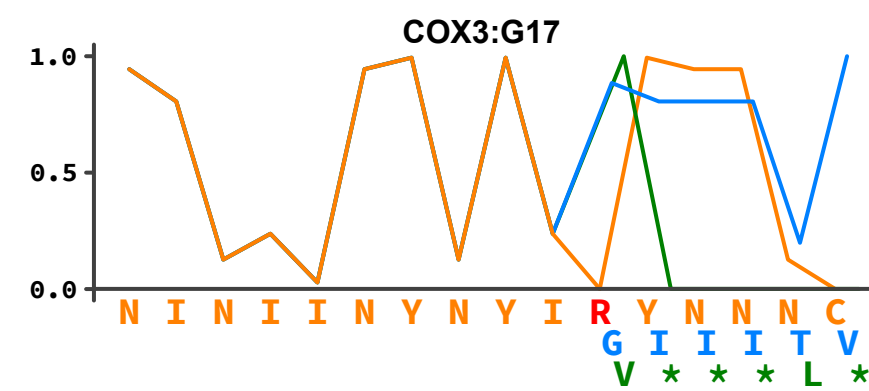

Reading frame 1 Reading frame 2 Reading frame 3

Supplement: msac191_Supplementary_Data [file msac191_supplementary_data.zip › Suppl Fig S10 - P atlanticus translatability at fs sites.pdf]

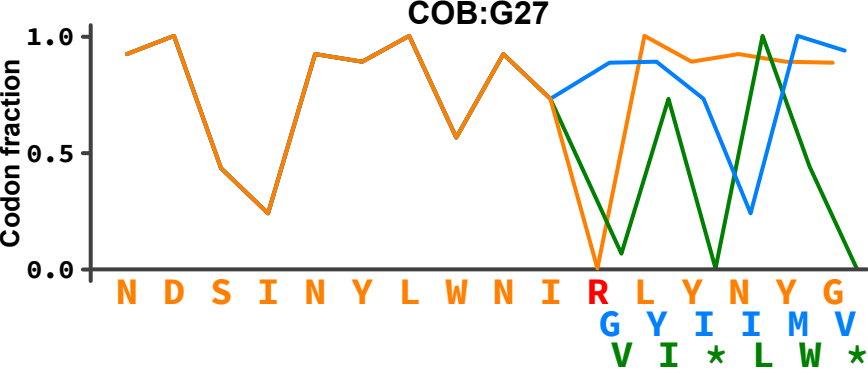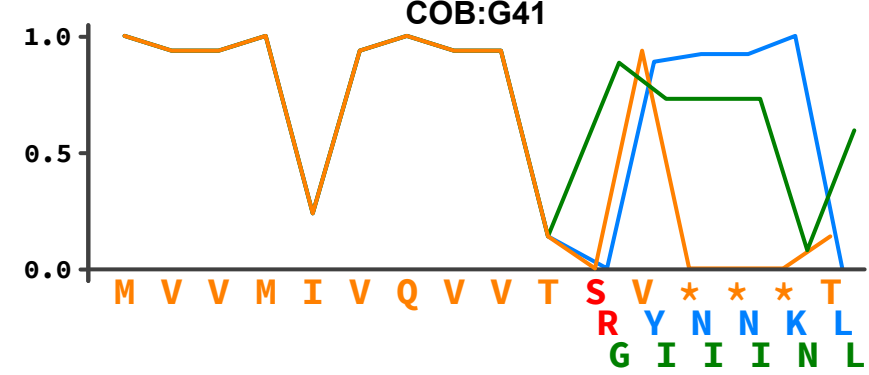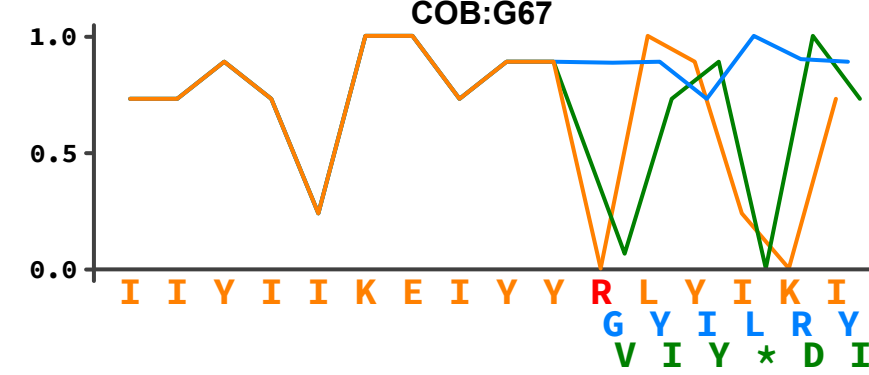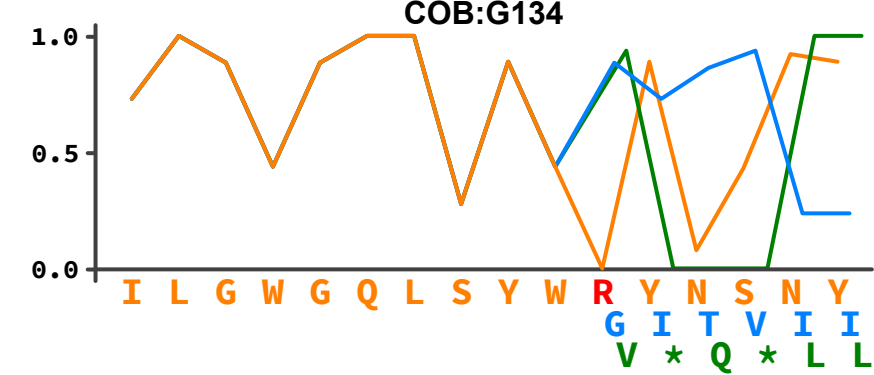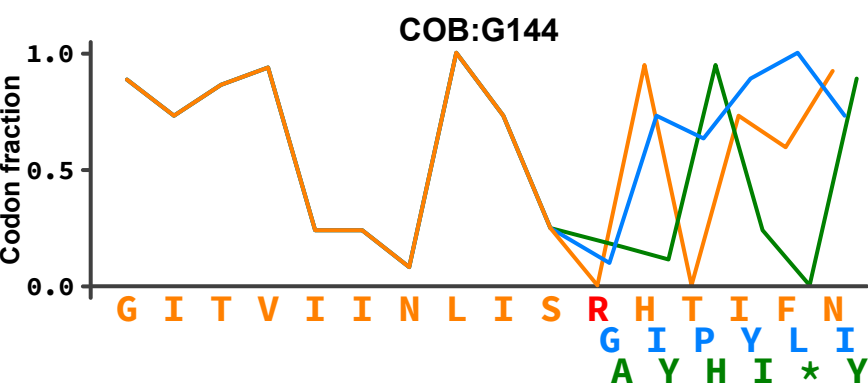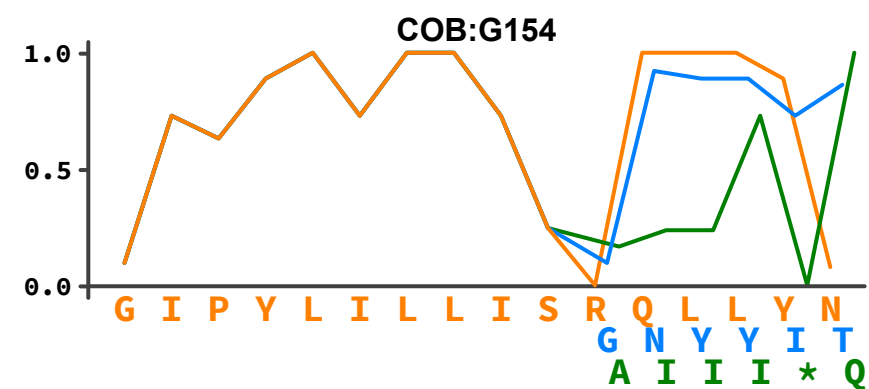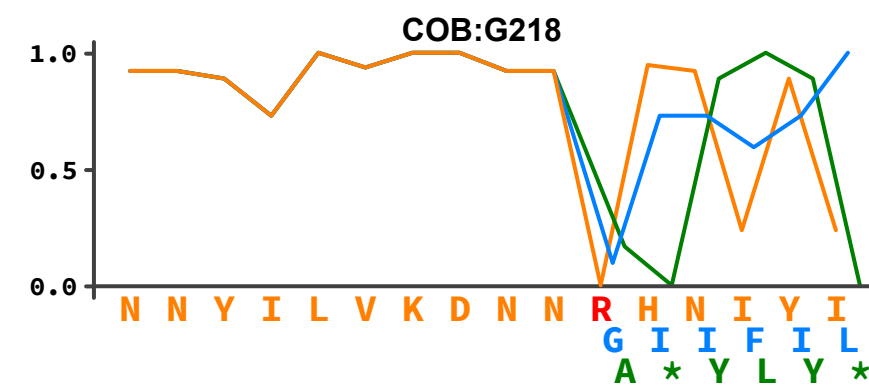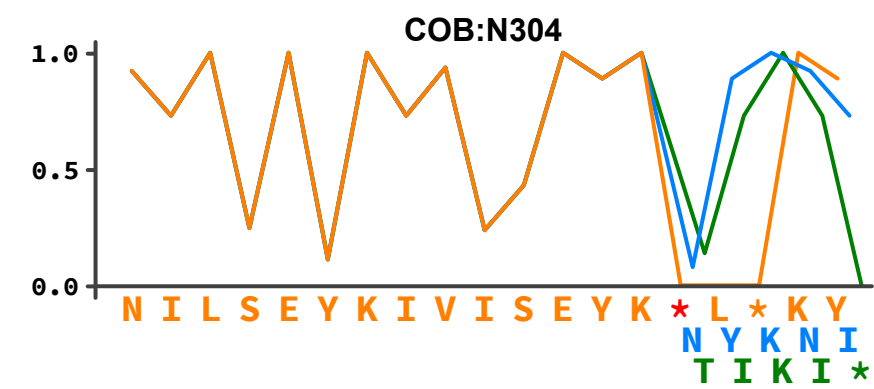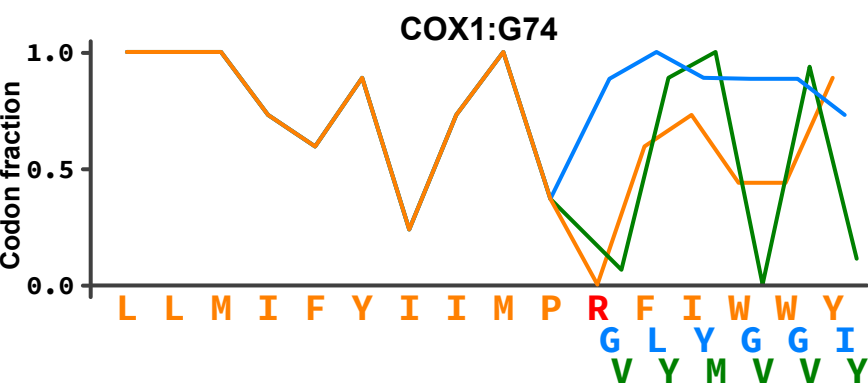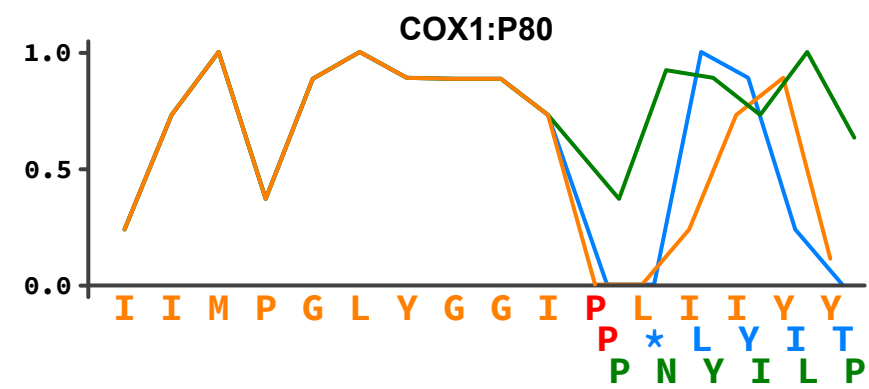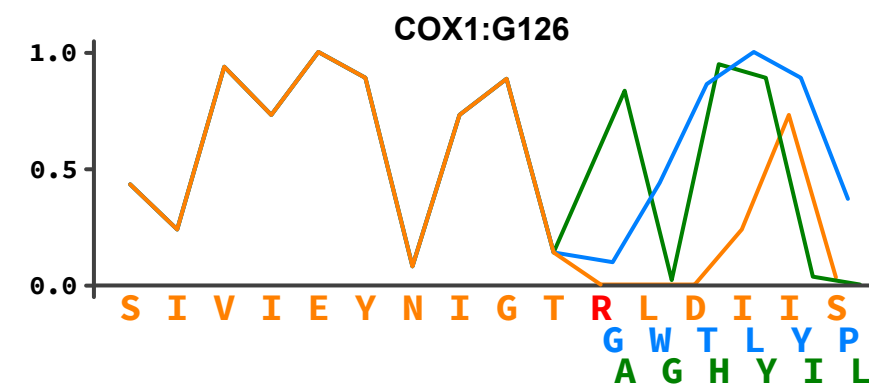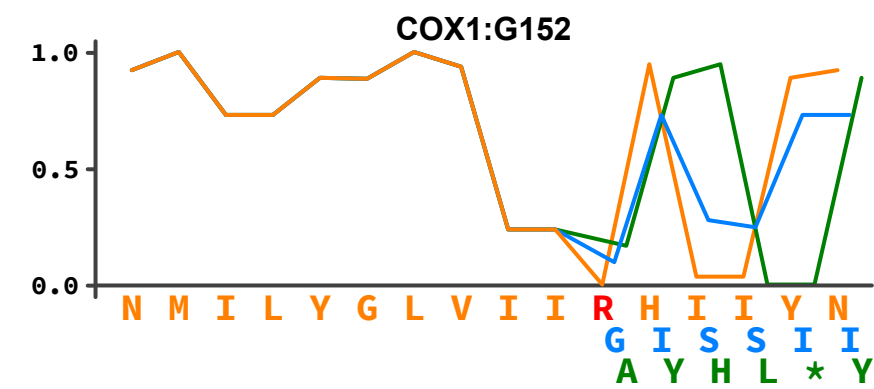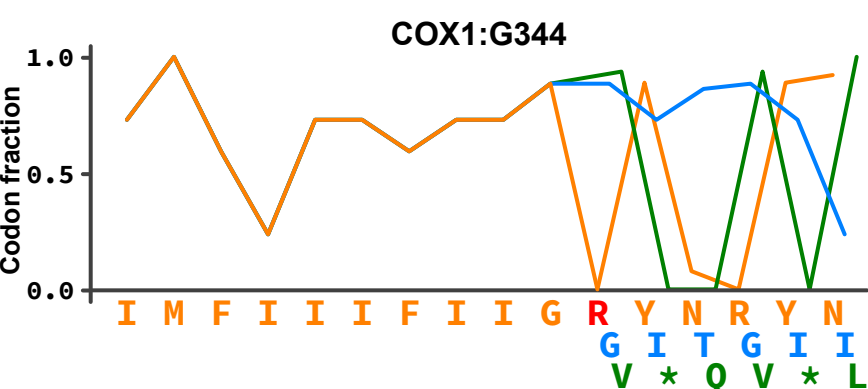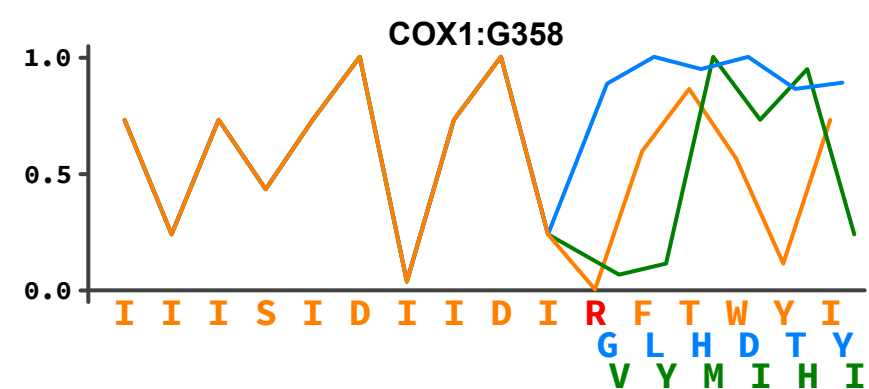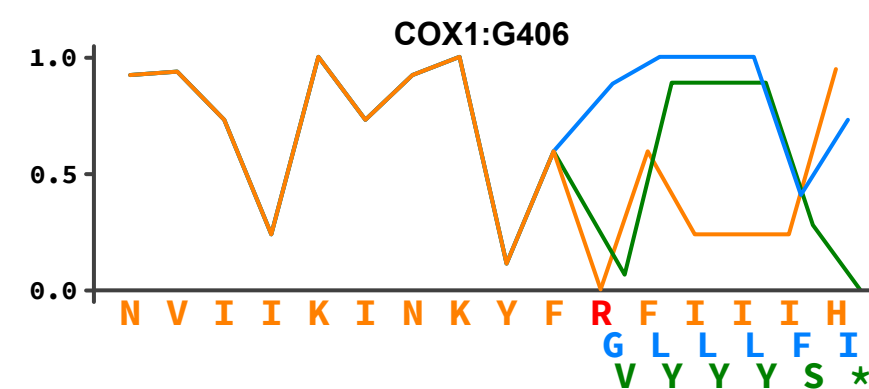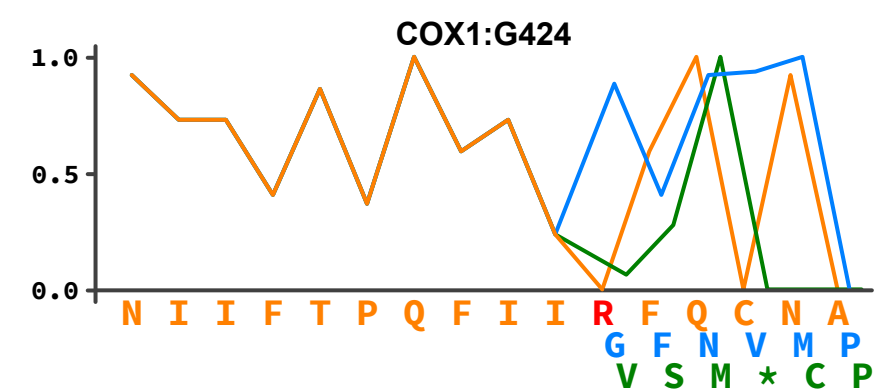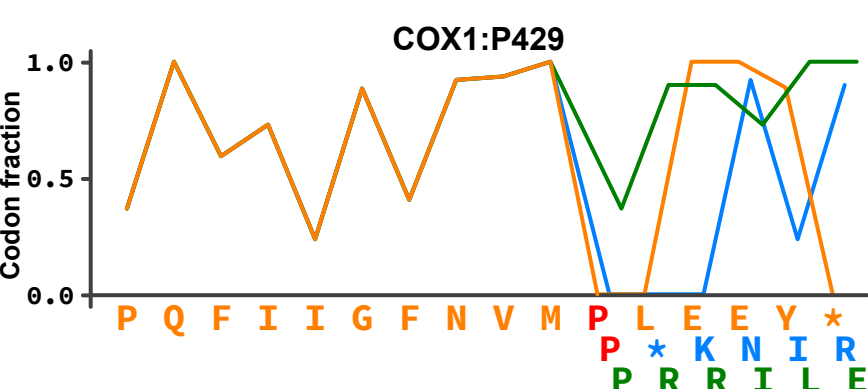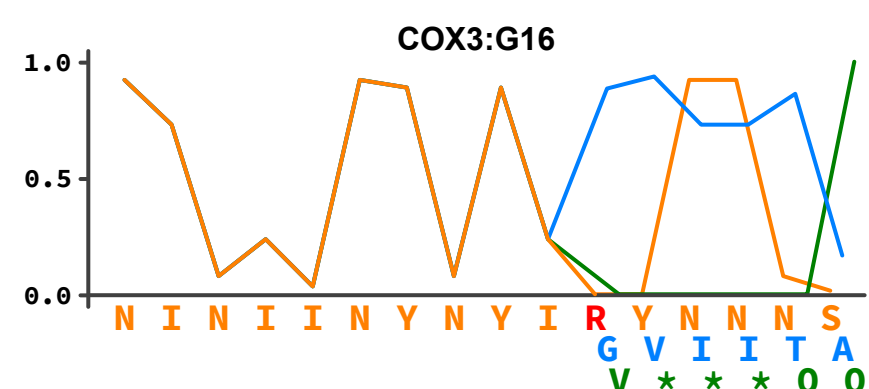

Reading frame 1 Reading frame 2 Reading frame 3

Supplement: msac191_Supplementary_Data [file msac191_supplementary_data.zip › Suppl Fig S11 - P chesapeaki translatability at fs sites.pdf]

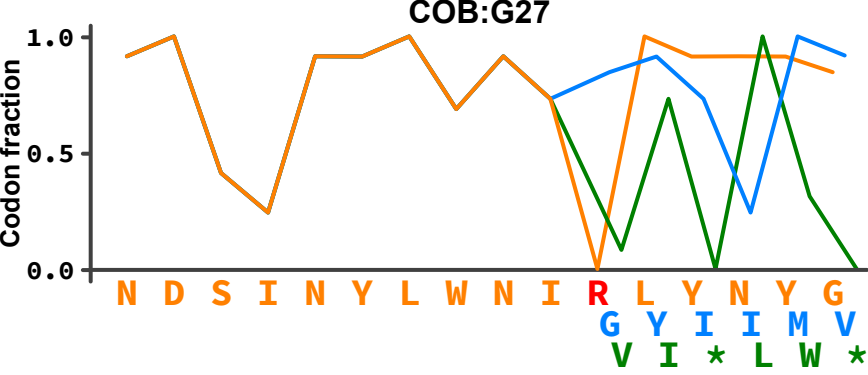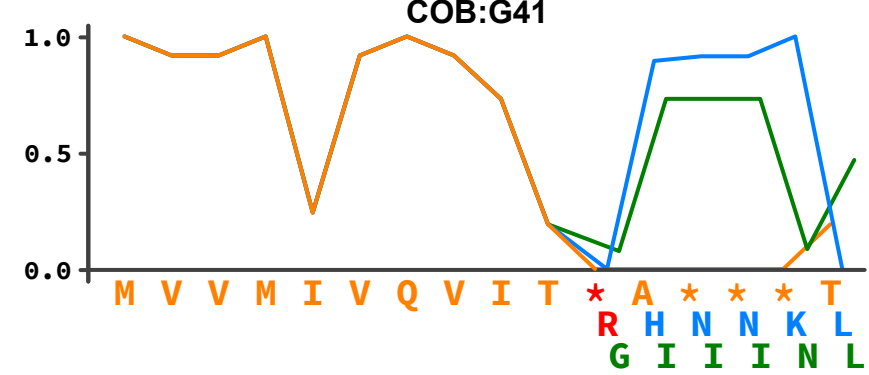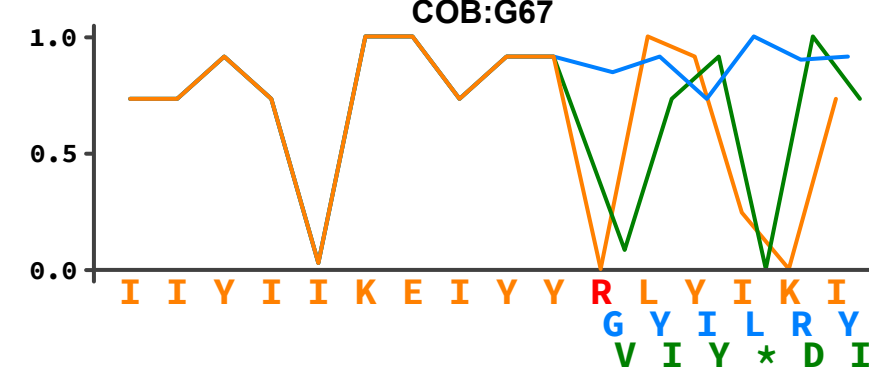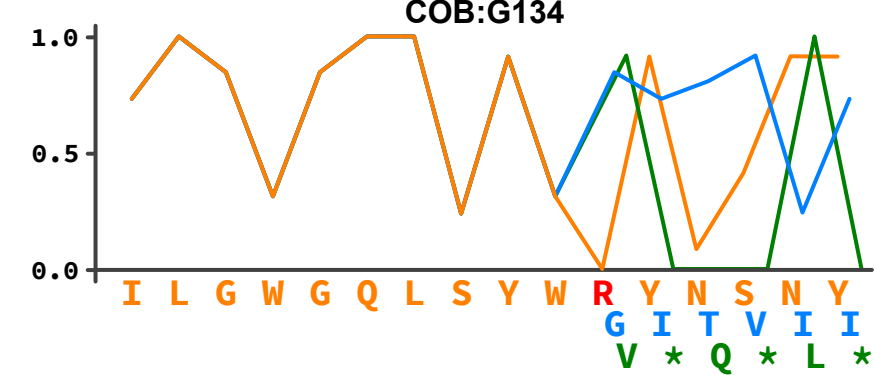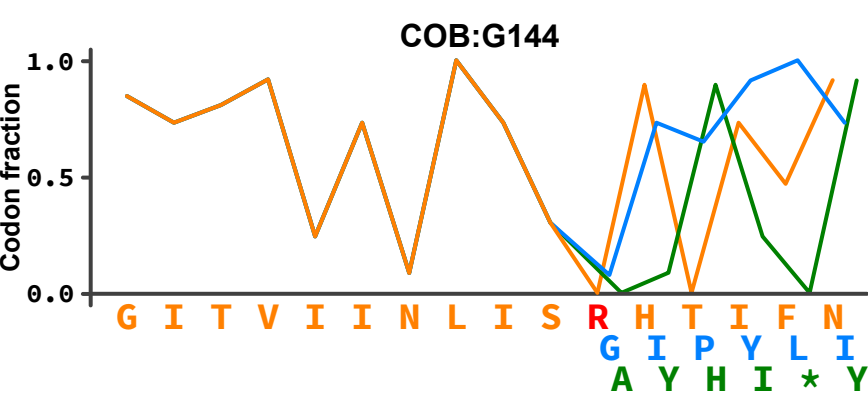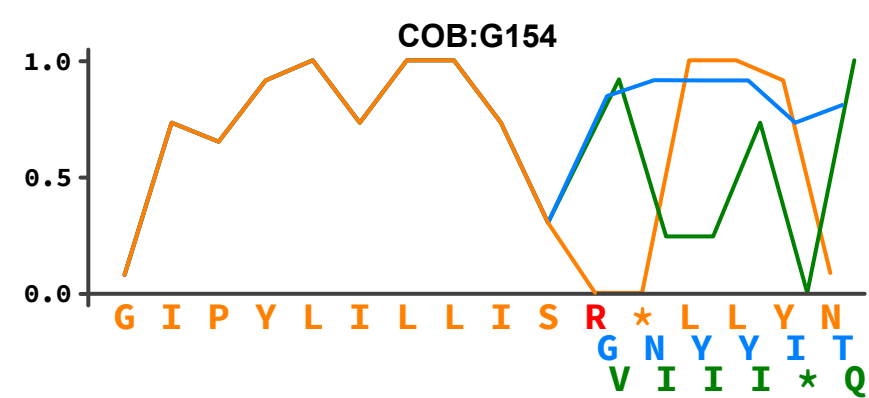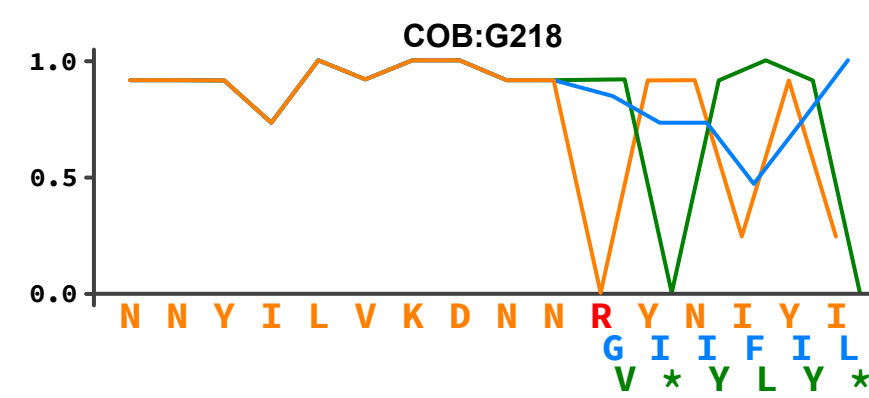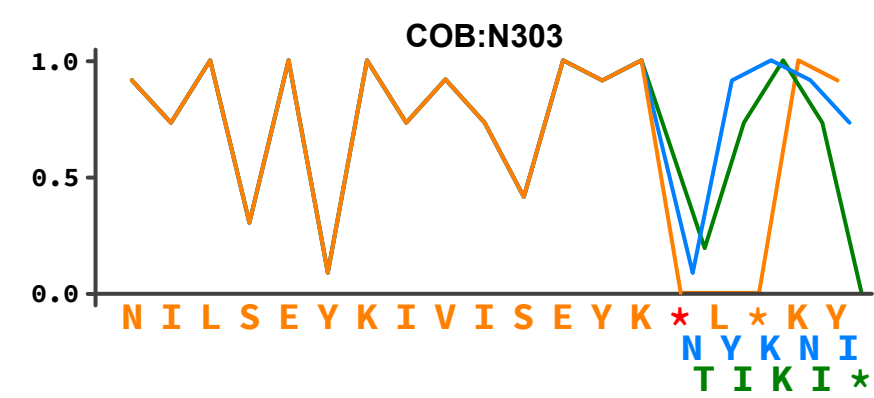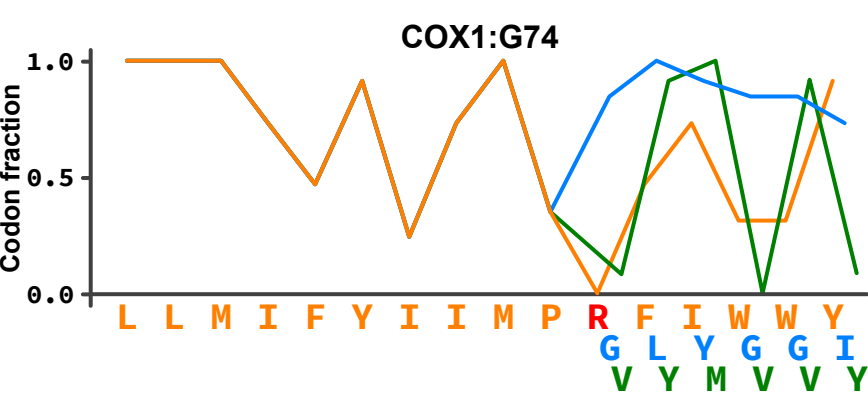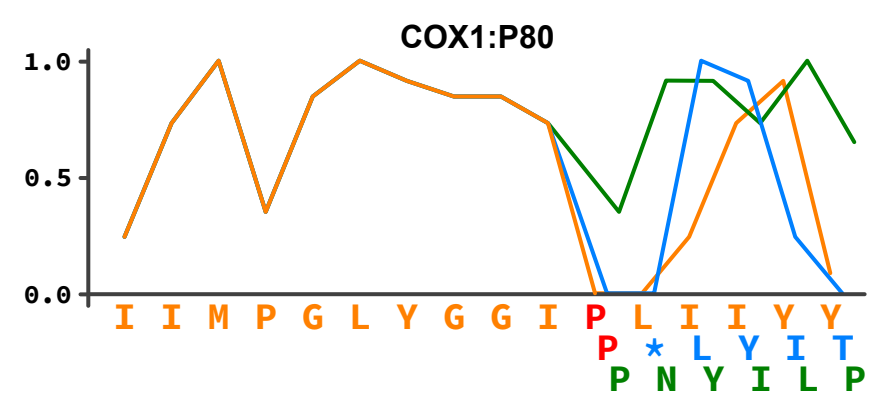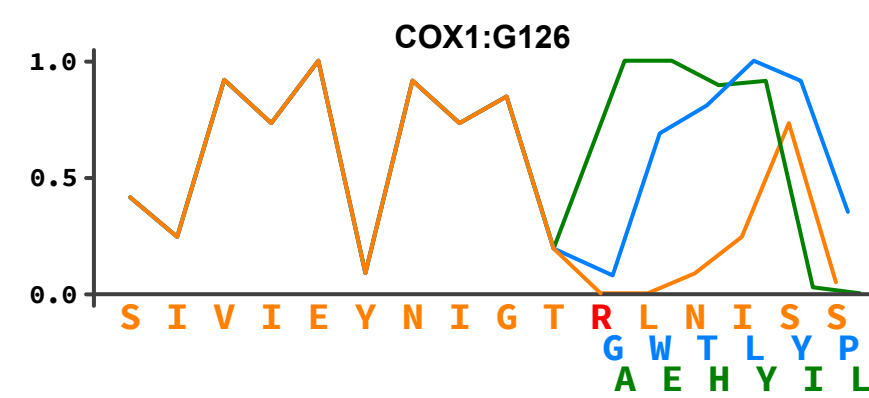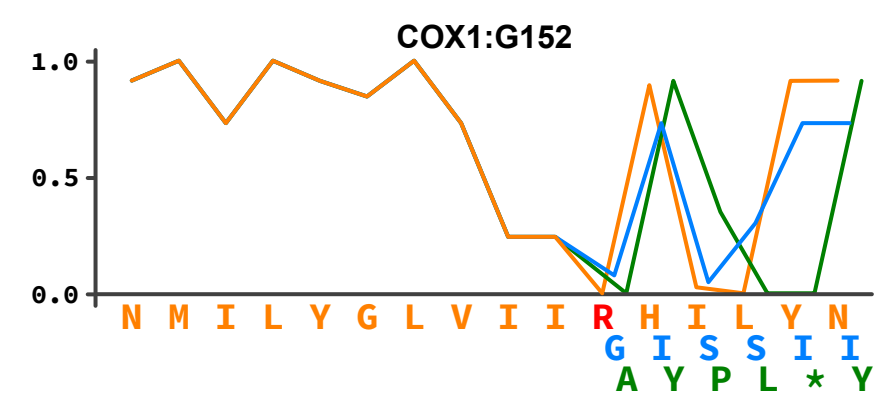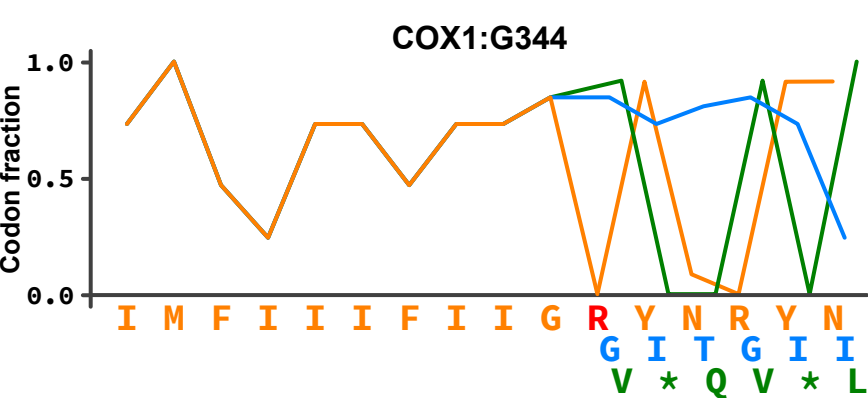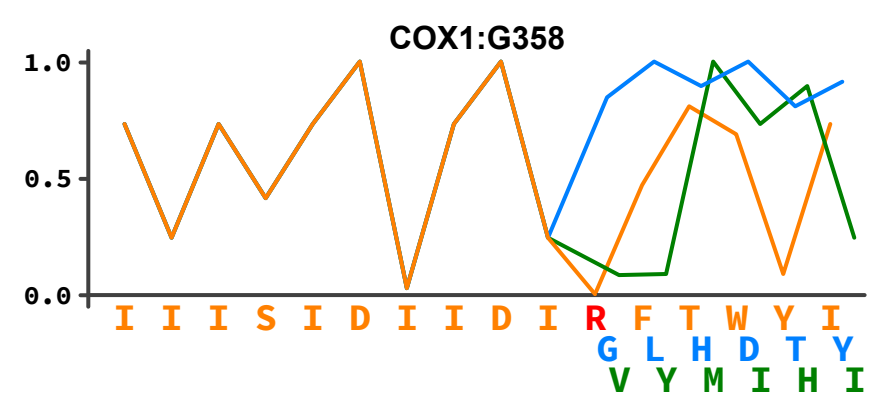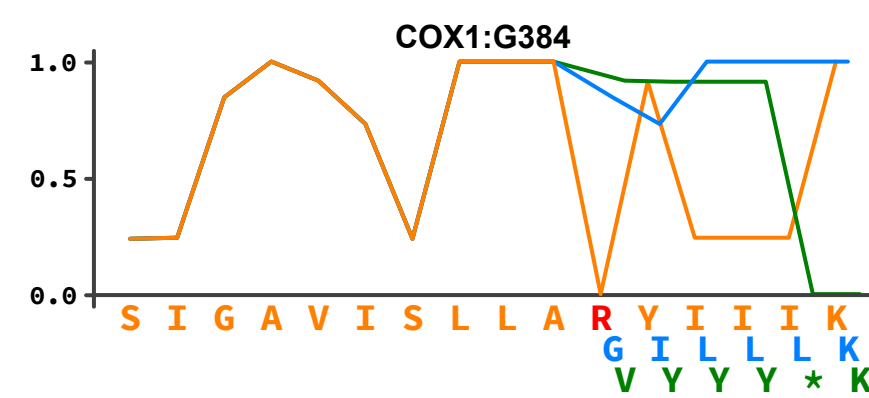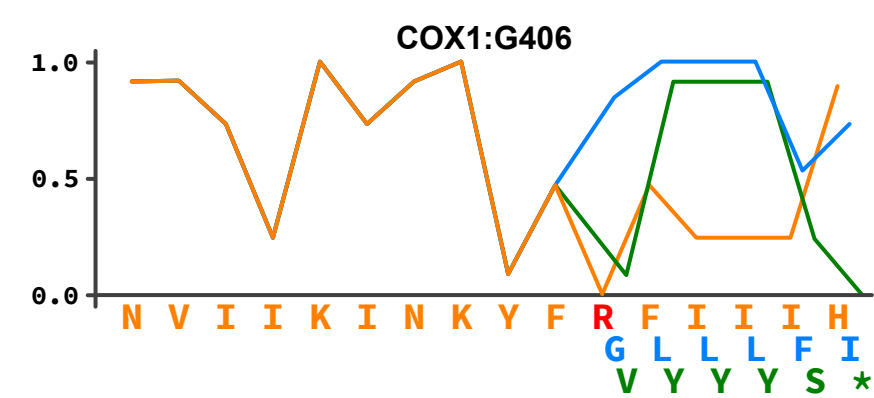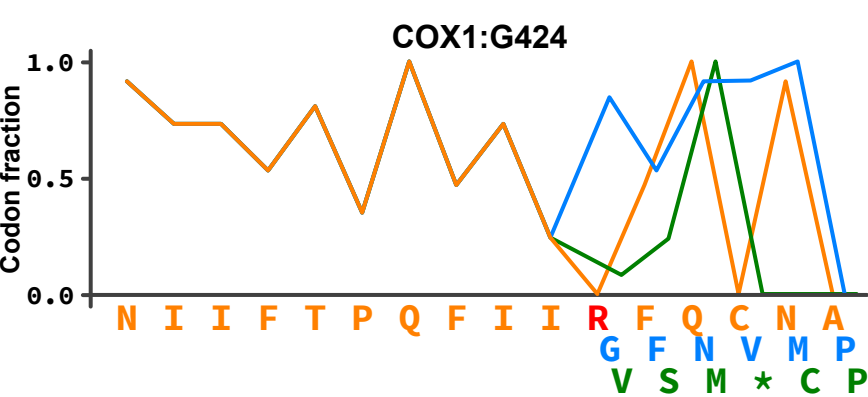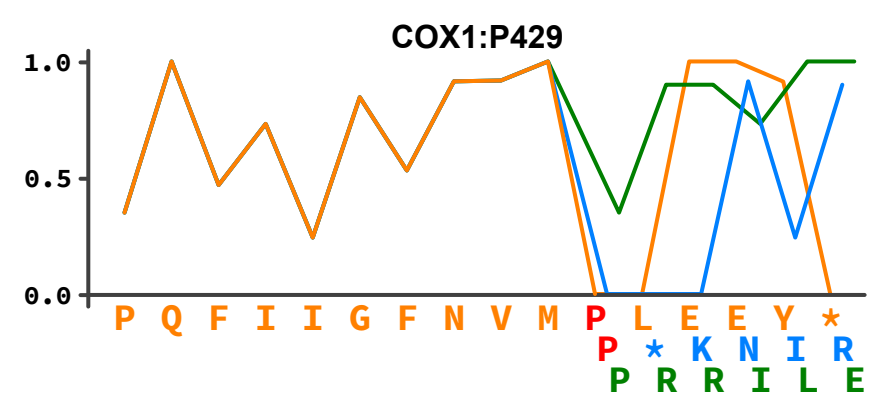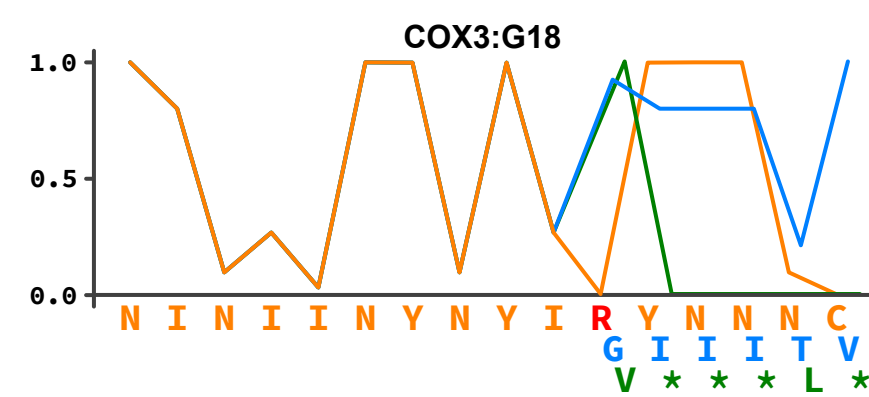

Reading frame 1 Reading frame 2 Reading frame 3

Supplement: msac191_Supplementary_Data [file msac191_supplementary_data.zip › Suppl Fig S12 - P marinus translatability at fs sites.pdf]

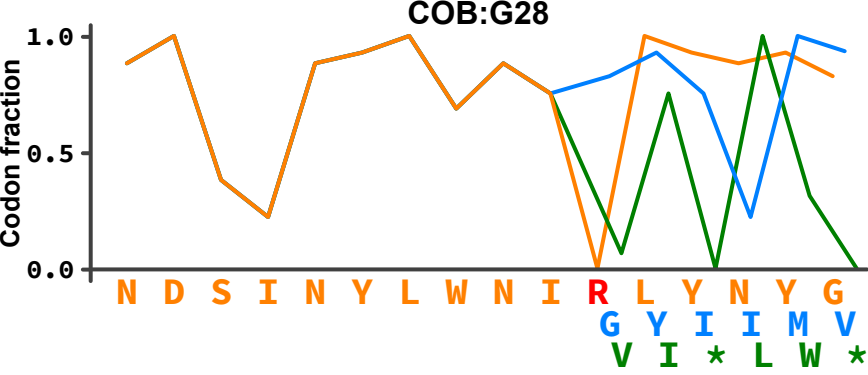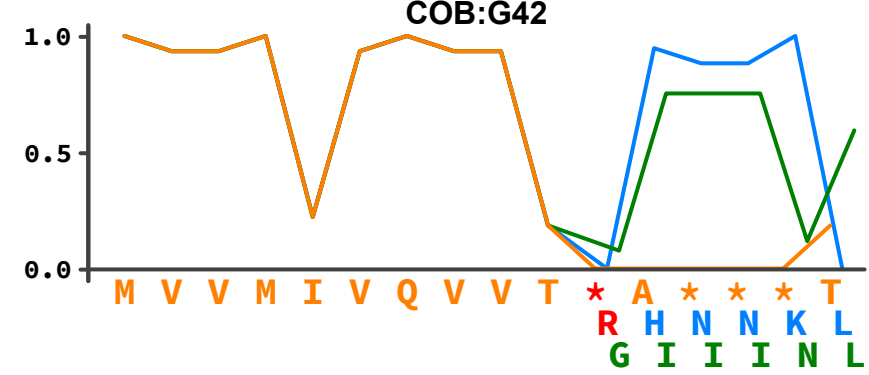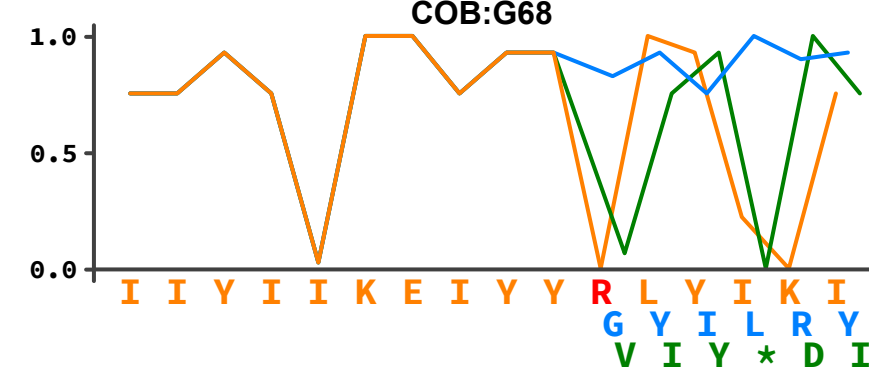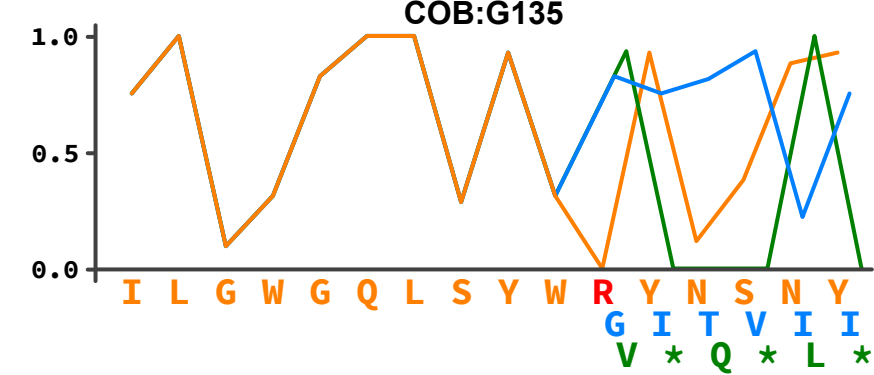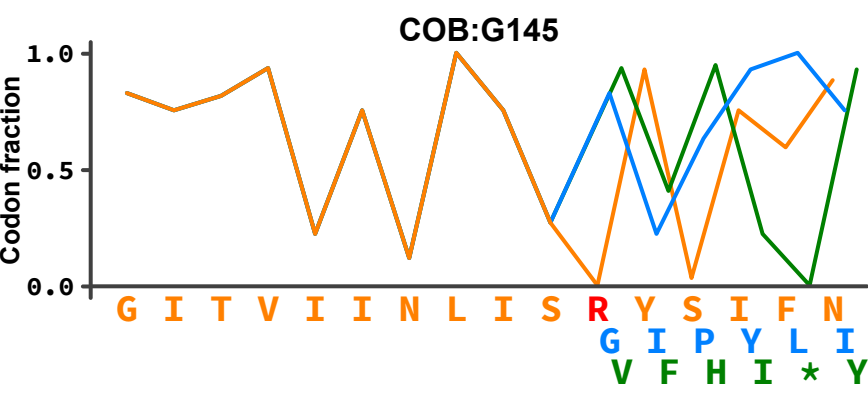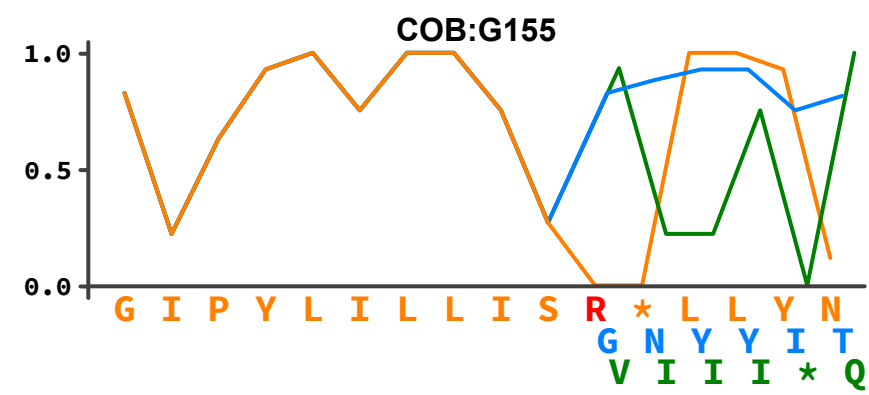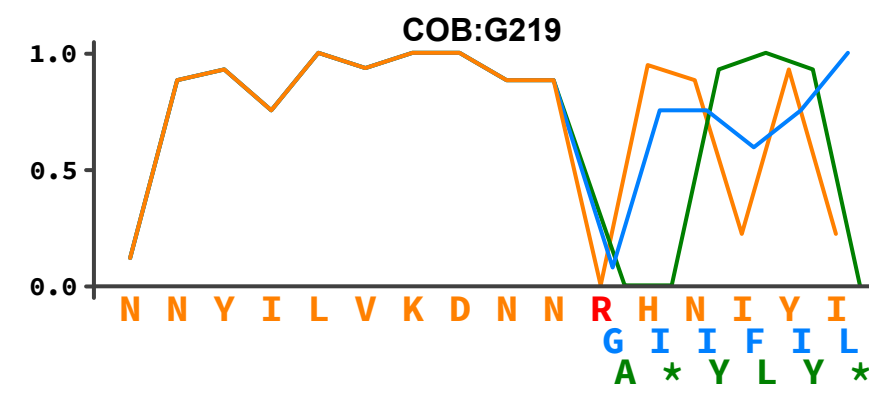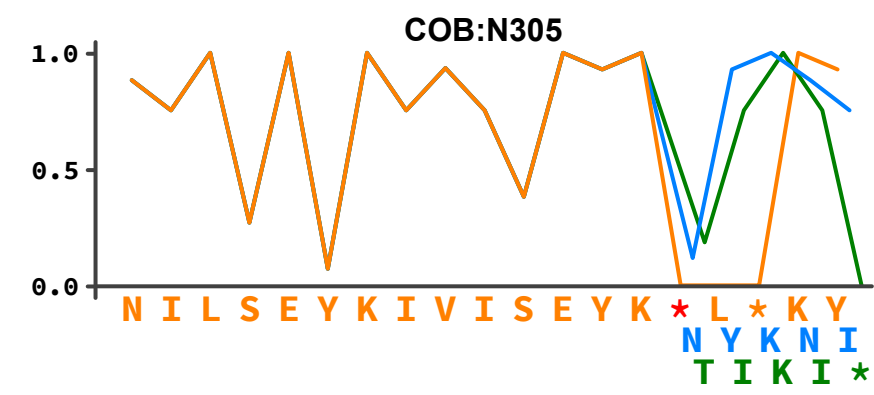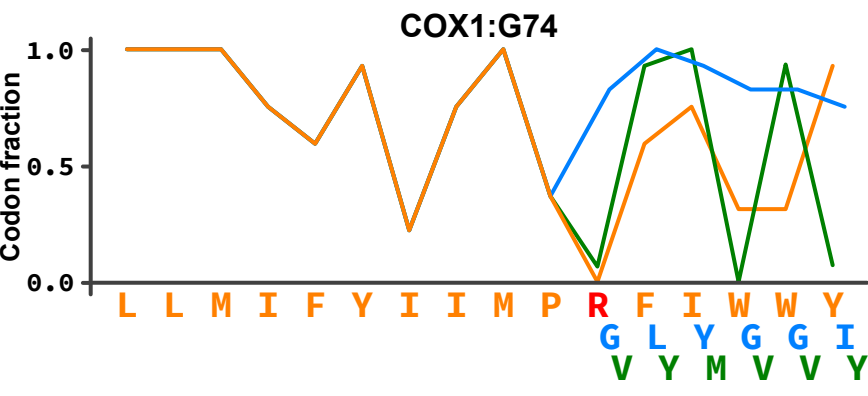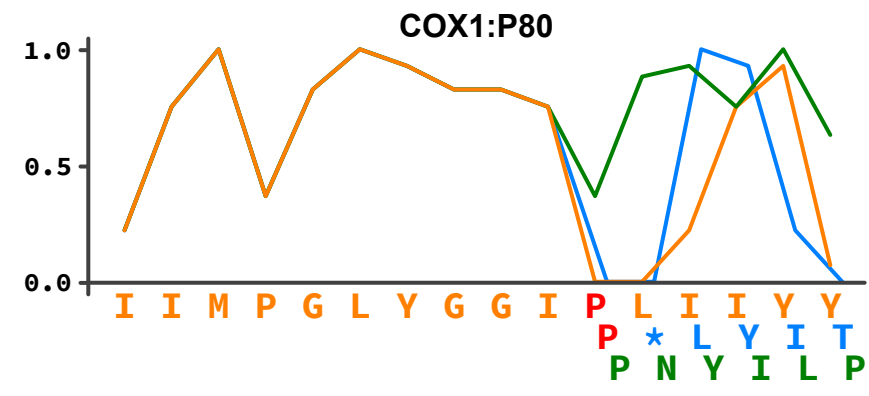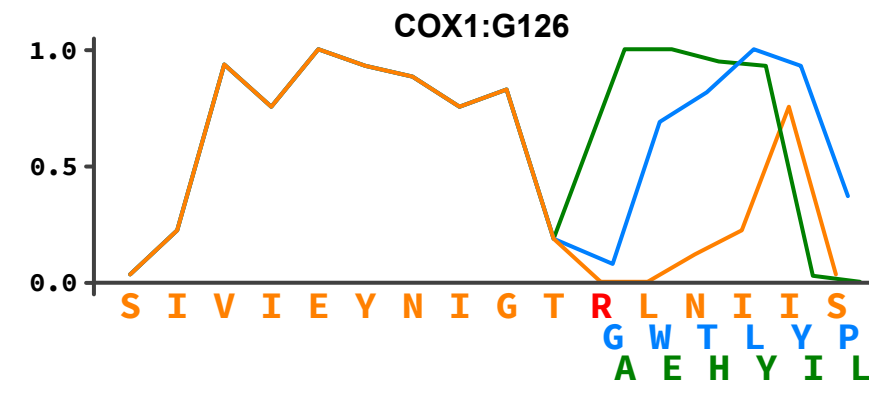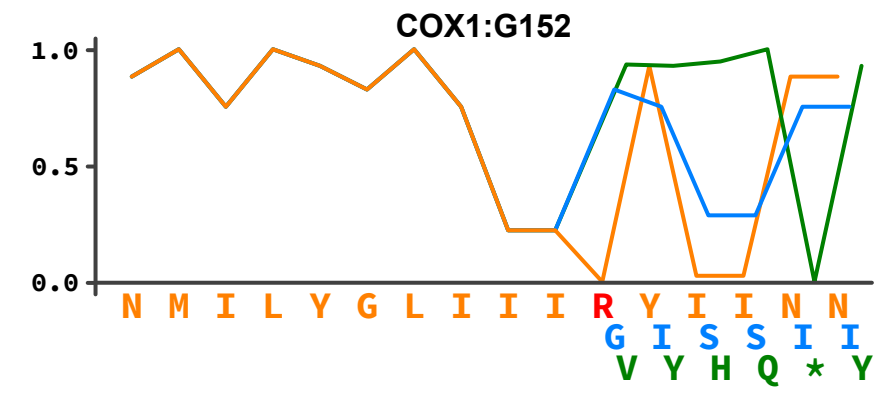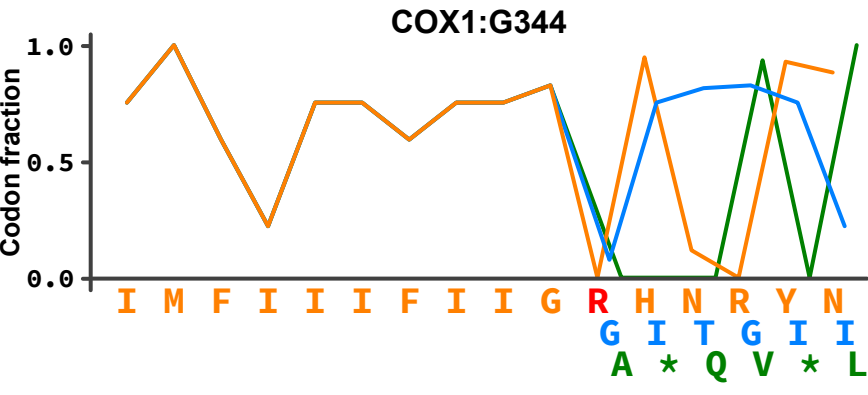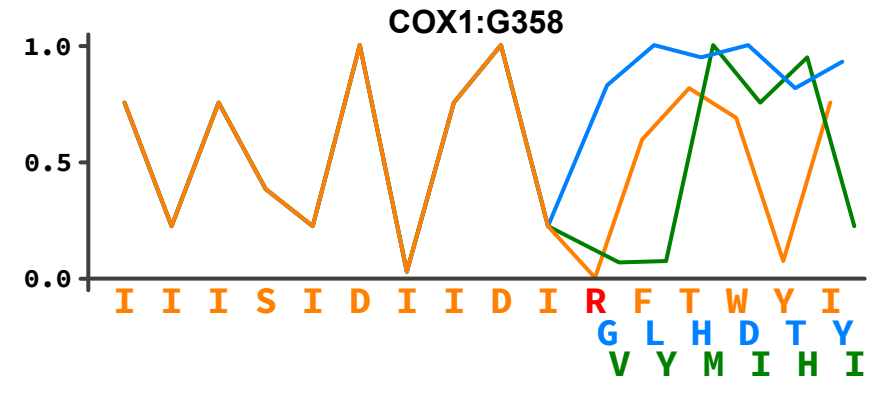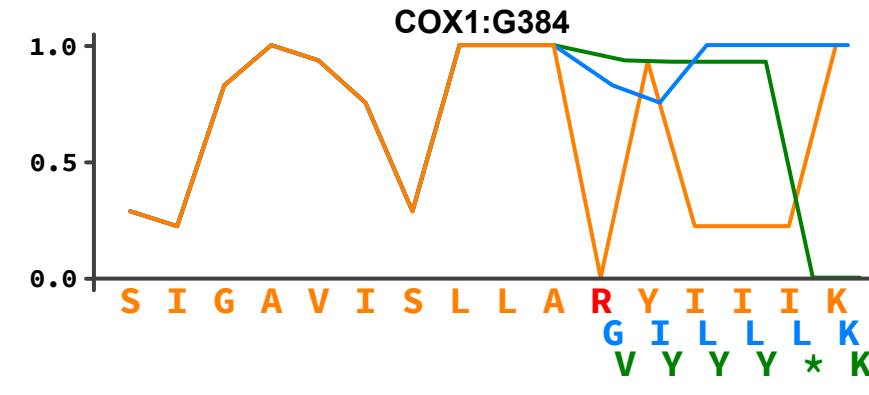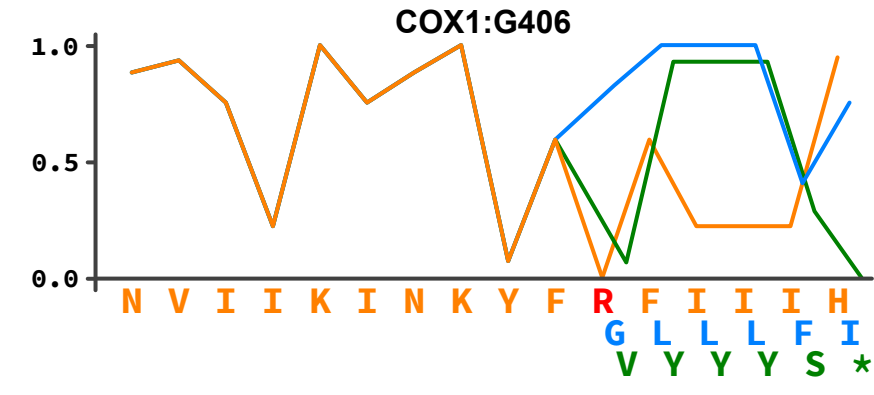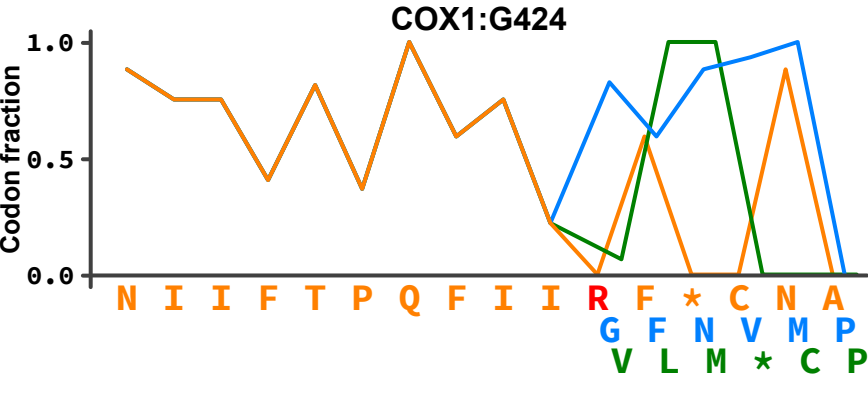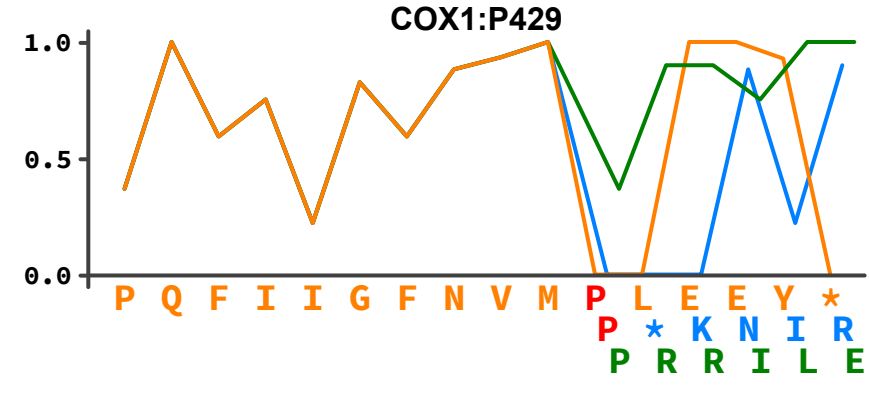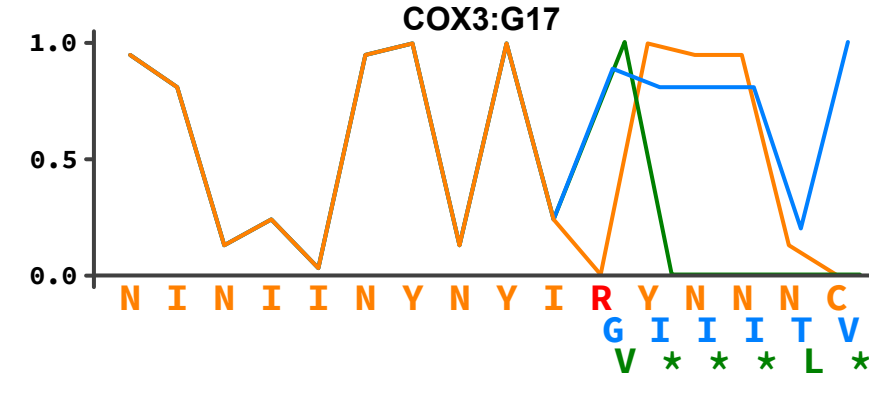

Reading frame 1 Reading frame 2 Reading frame 3

Supplement: msac191_Supplementary_Data [file msac191_supplementary_data.zip › Suppl Fig S13 - P olseni translatability at fs sites.pdf]

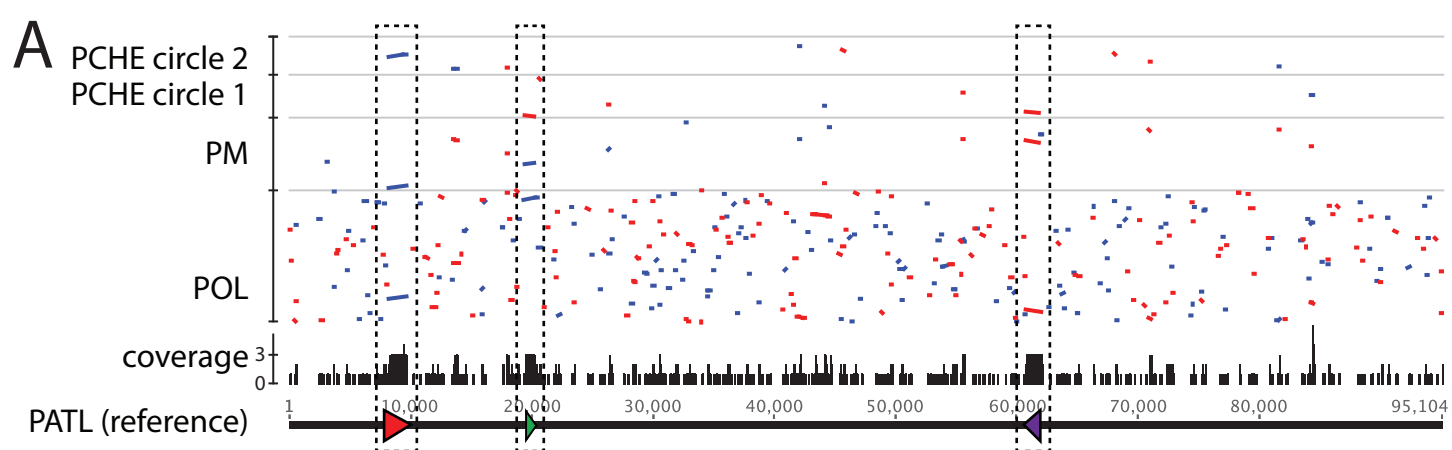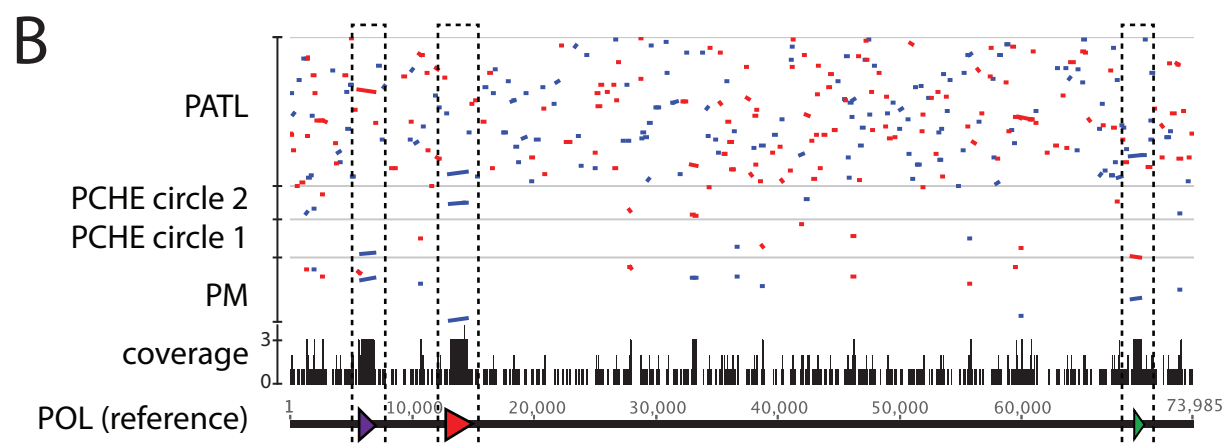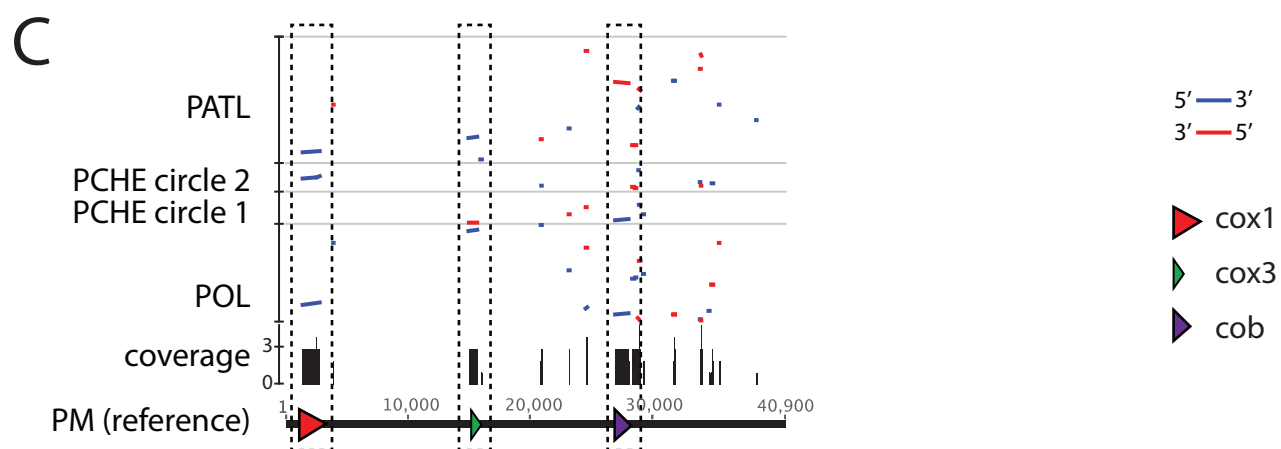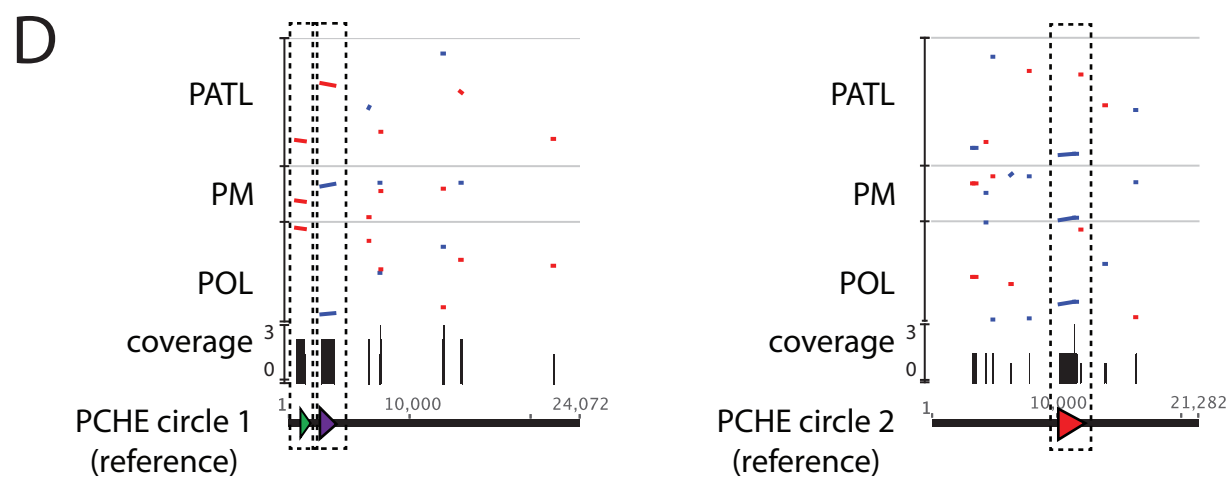

Supplement: msac191_Supplementary_Data [file msac191_supplementary_data.zip › Suppl Fig S2 - LASTZ alignments.pdf]

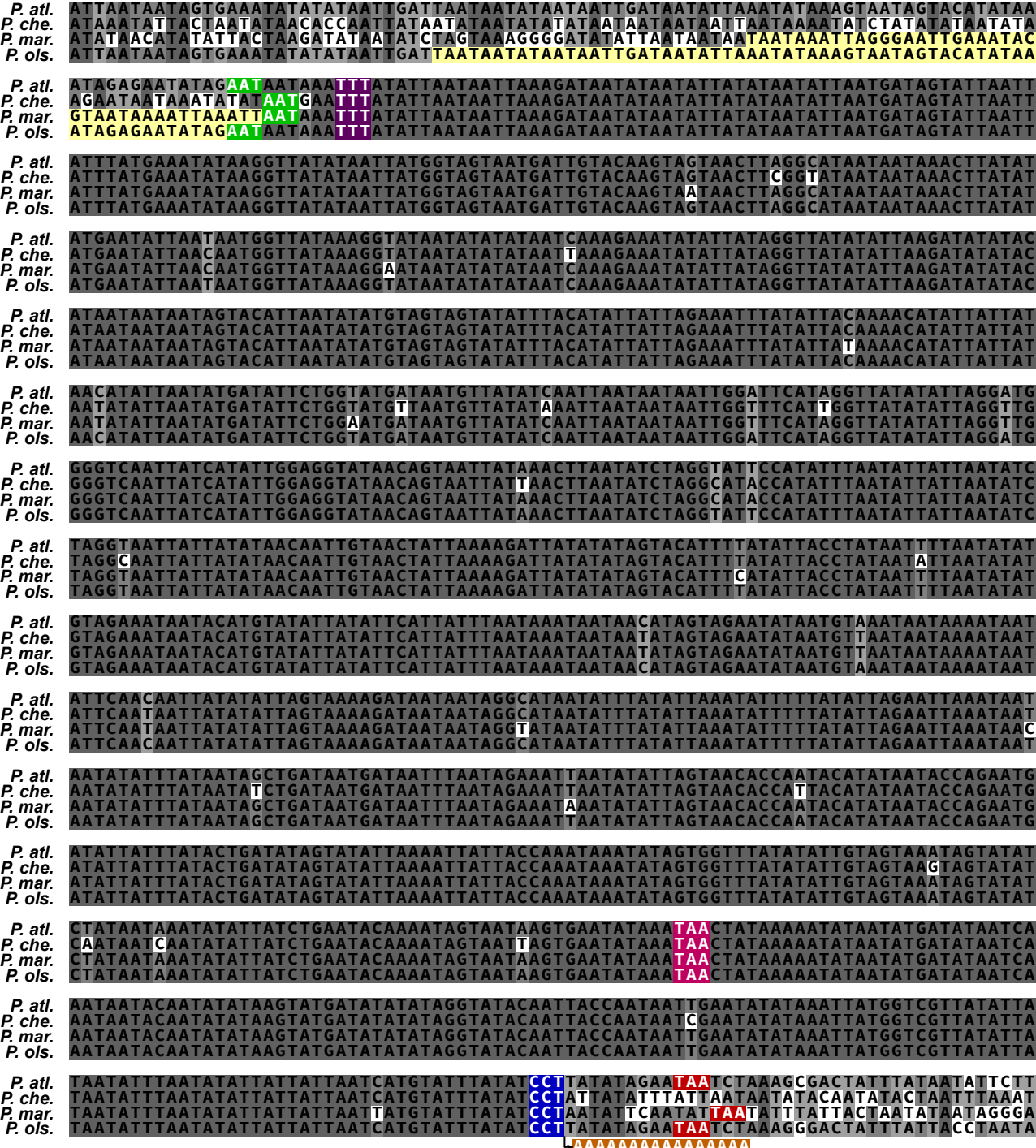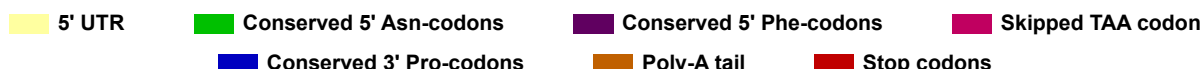

Supplement: msac191_Supplementary_Data [file msac191_supplementary_data.zip › Suppl Fig S3 - cob full alignment.pdf]

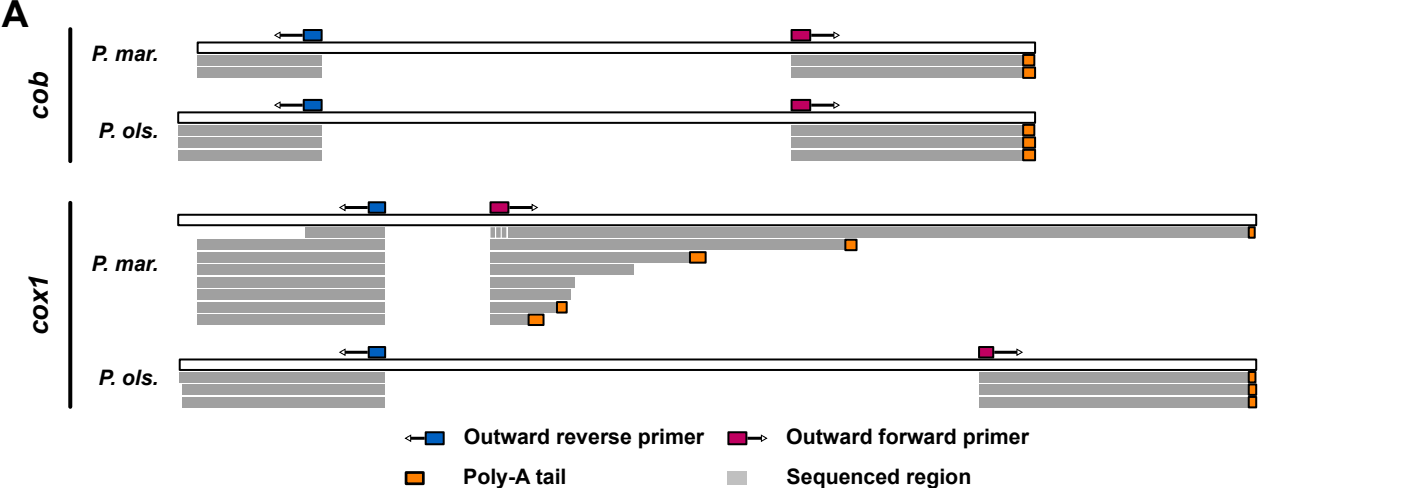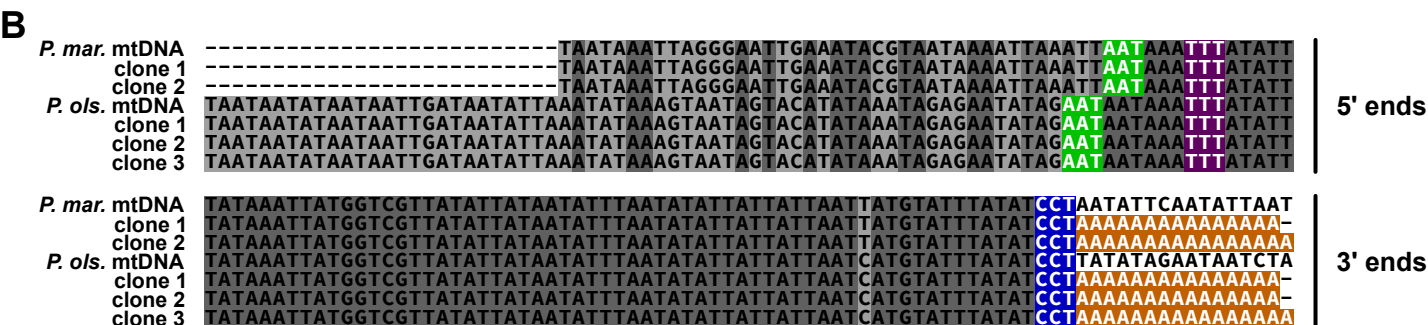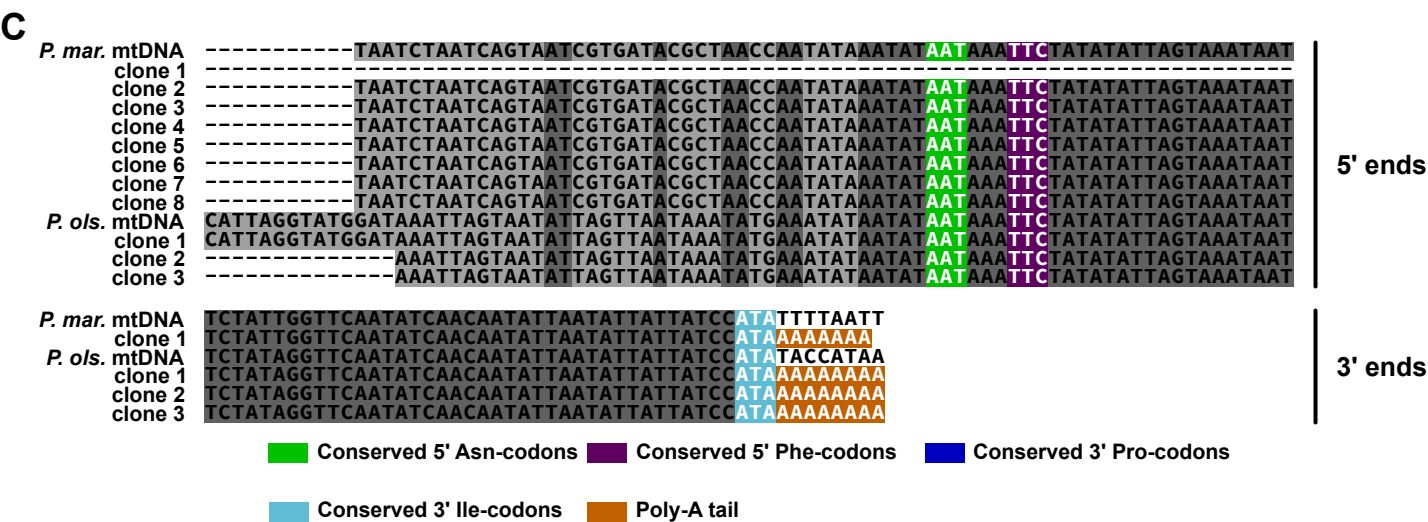

Supplement: msac191_Supplementary_Data [file msac191_supplementary_data.zip › Suppl Fig S6 - cRT-PCR schematic and results.pdf]

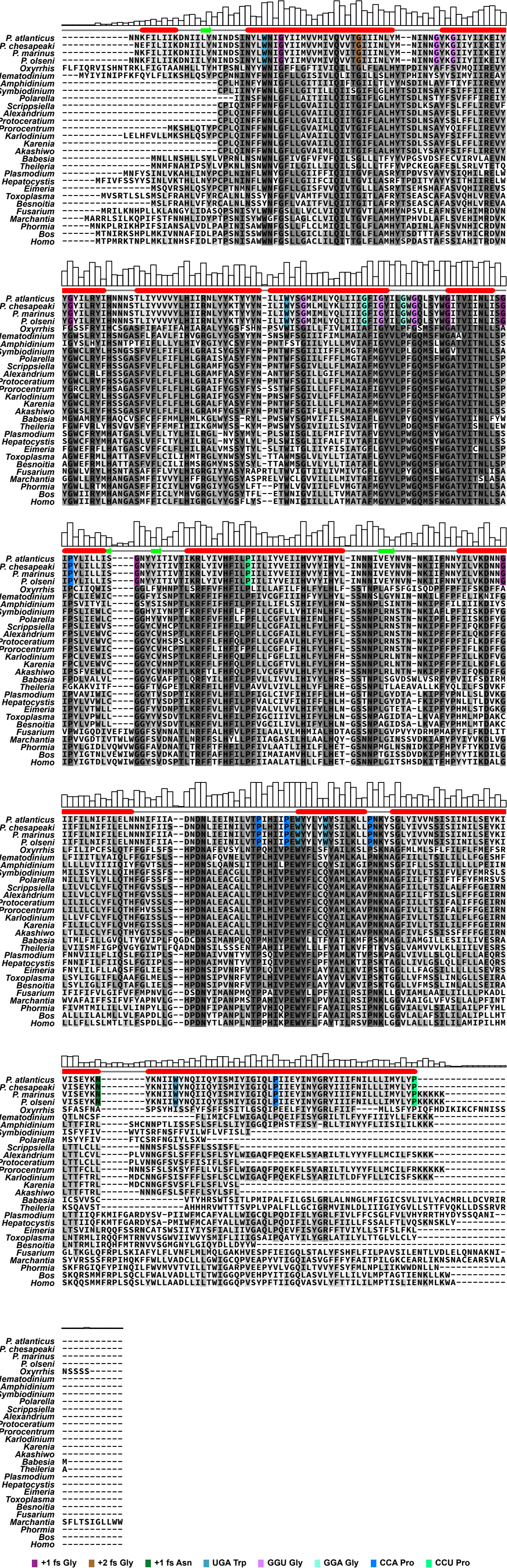

Supplement: msac191_Supplementary_Data [file msac191_supplementary_data.zip › Suppl Fig S7 - cob protein alignment.pdf]

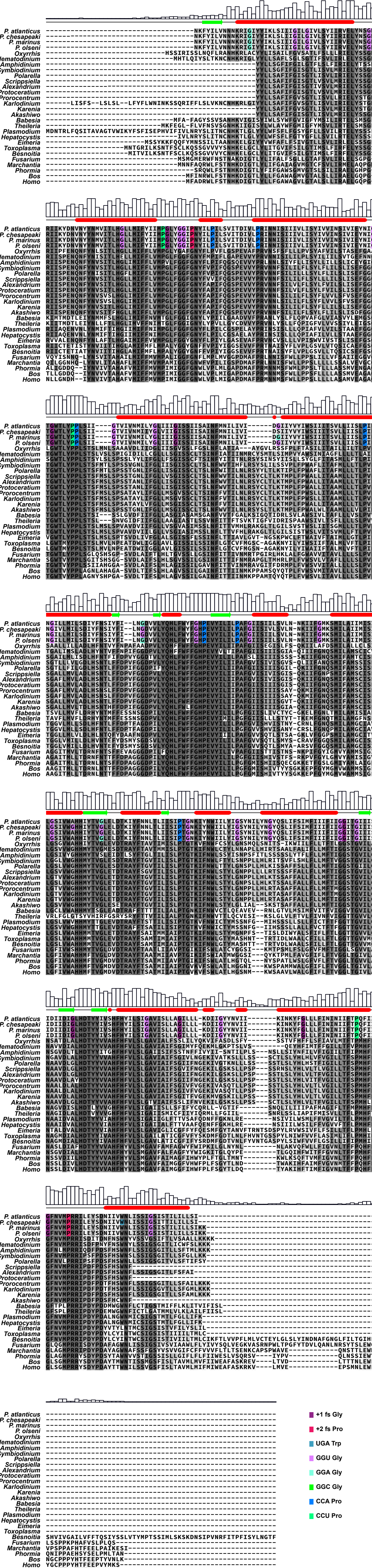

Supplement: msac191_Supplementary_Data [file msac191_supplementary_data.zip › Suppl Fig S8 - cox1 protein alignment.pdf]

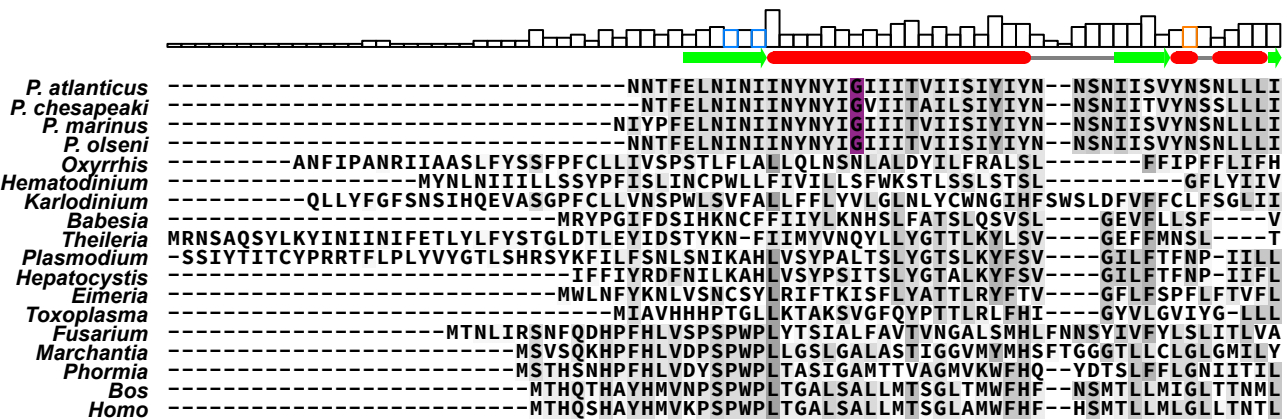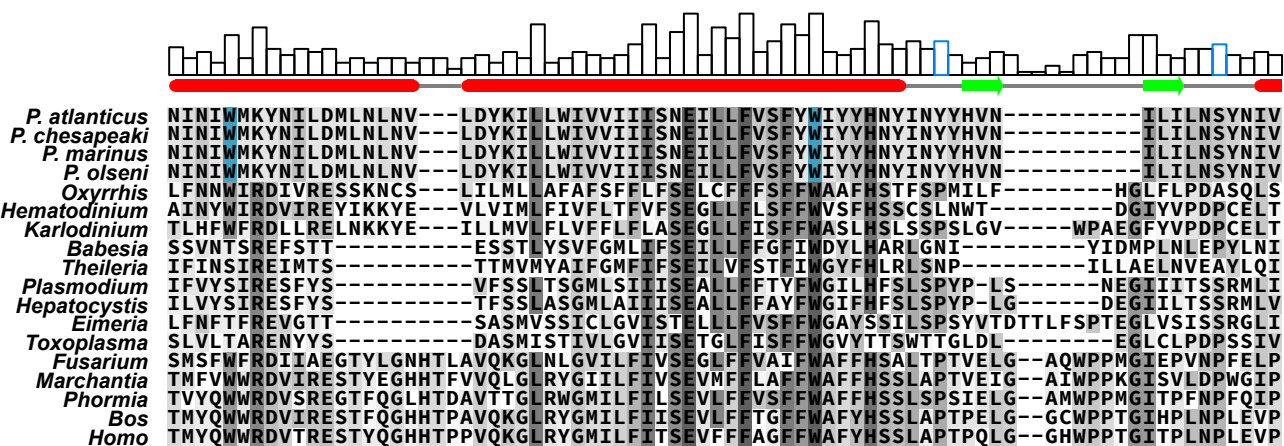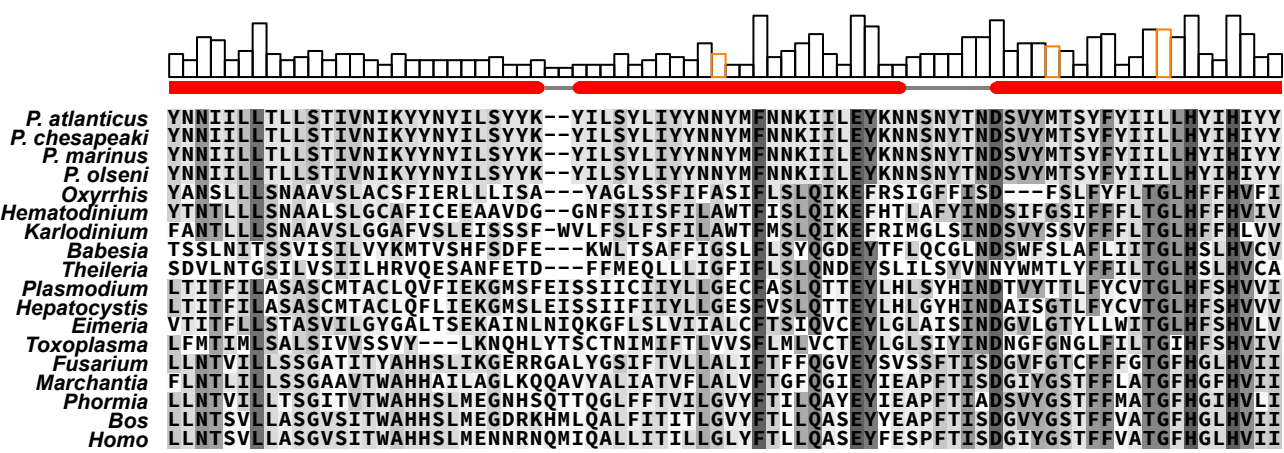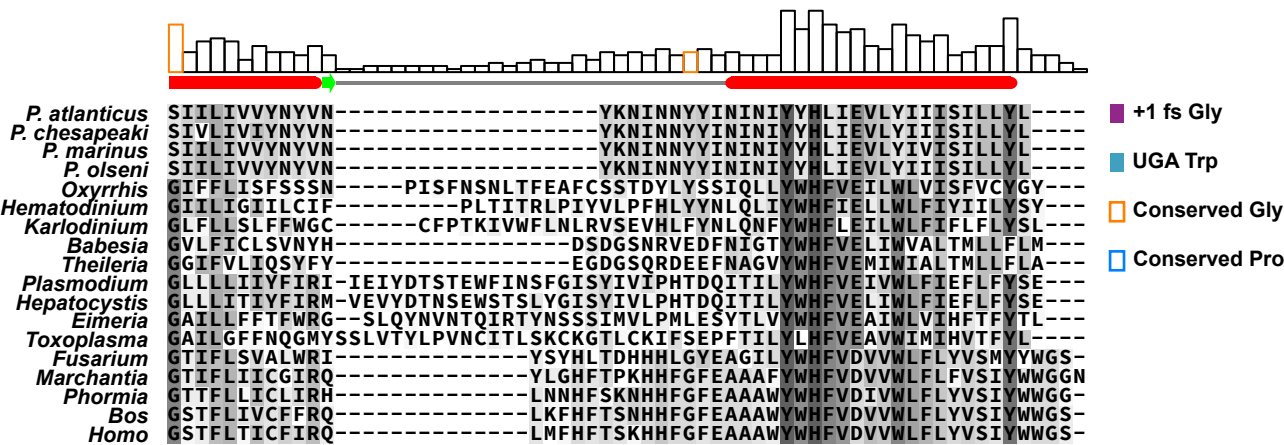

Supplement: msac191_Supplementary_Data [file msac191_supplementary_data.zip › Suppl Fig S9 - cox3 protein alignment.pdf]
