# Supplementary material for: Mitochondrial Genomes in Perkinsus Decode Conserved Frameshifts in All Genes
Source: Mol Biol Evol. 2022 Sep 15;39(10):msac191. doi: 10.1093/molbev/msac191 (PMC9550989; doi:10.1093/molbev/msac191)
Supplement: msac191_Supplementary_Data [file Suppl Fig S4 - cox1 full alignment.pdf › Suppl Fig S4 - cox1 full alignment.pdf]

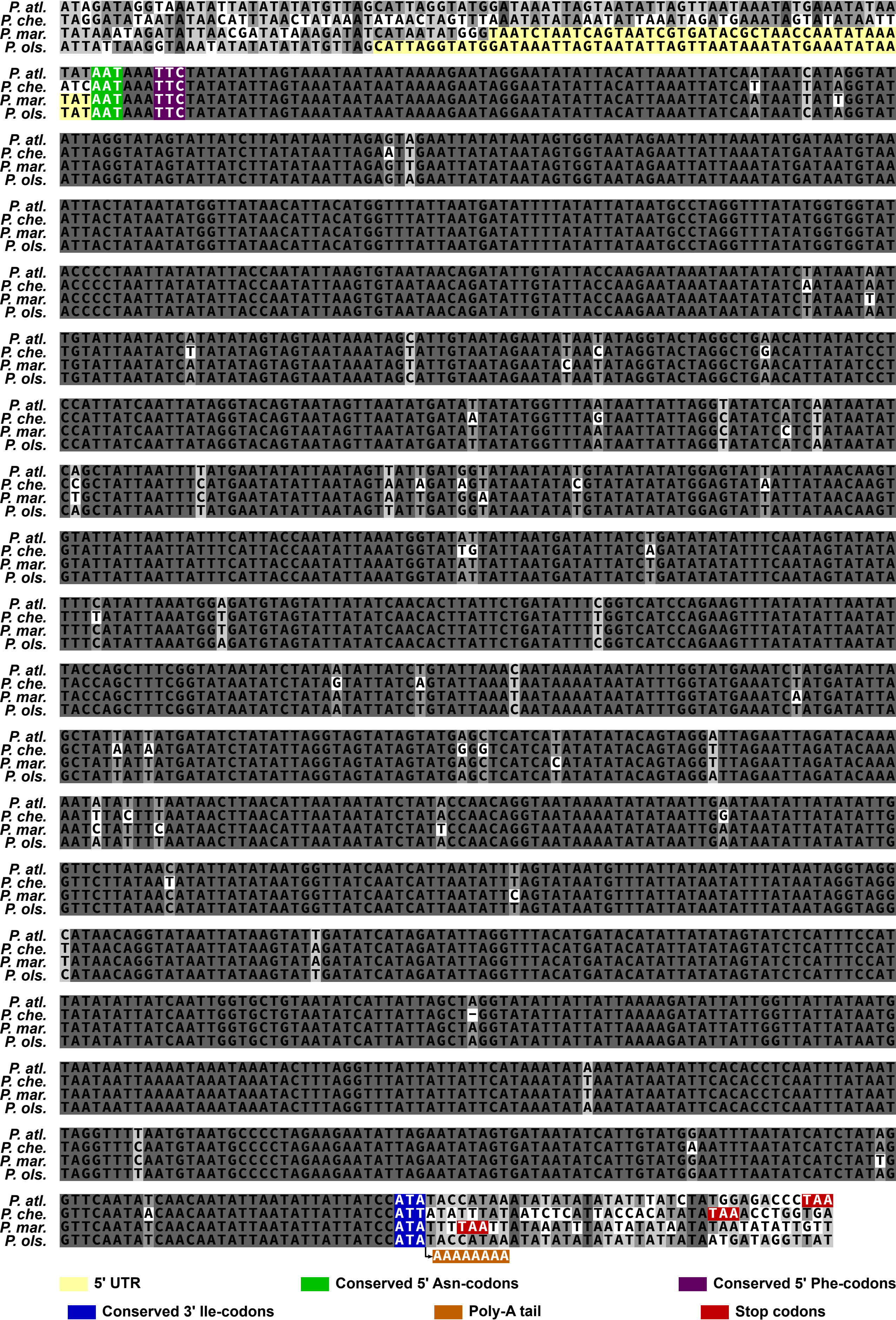
P. atl.
P. che.
P. mar.
P. ols.
AAAAAAAA
5' UTR
Conserved 5' Asn-codons
Conserved 5' Phe-codons
Conserved 3' Ile-codons
Poly-A tail
Stop codons
