# Supplementary material for: Mitochondrial Genomes in Perkinsus Decode Conserved Frameshifts in All Genes
Source: Mol Biol Evol. 2022 Sep 15;39(10):msac191. doi: 10.1093/molbev/msac191 (PMC9550989; doi:10.1093/molbev/msac191)
Supplement: msac191_Supplementary_Data [file Suppl Fig S5 - cox3 full alignment.pdf › Suppl Fig S5 - cox3 full alignment.pdf]

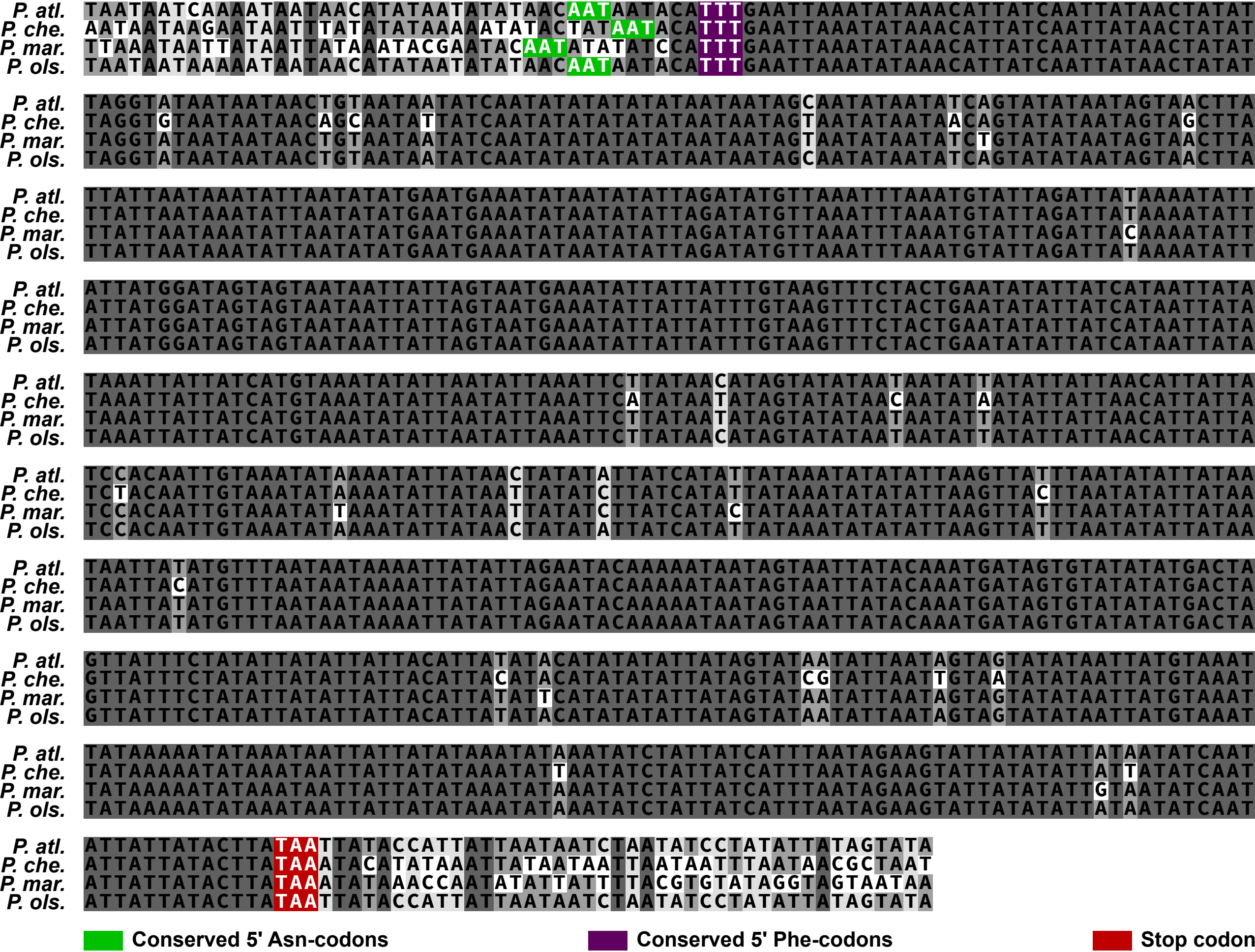
P. atl. TAATAATCAAAATAATAACATATAATATATAACAATAATACATTTGAATTAAATATAAACATTATCAATTATAACTATAT
P. che. AATAATAAGAATAATTTATATATAAAAATATACTATAATACATTTGAATTAAATATAAACATTATCAATTATAACTATAT
P. mar. TTAAATAATTATAATTATAAATACGAATACAATATATATCCATTTGAATTAAATATAAACATTATCAATTATAACTATAT
P. ols. TAATAATAAAAATAATAACATATAATATATAACAATAATACATTTGAATTAAATATAAACATTATCAATTATAACTATAT
P. atl. TAGGTATAATAATAACTGTAATAATATCAATATATATATATAATAATAGCAATATAATATCAGTATATAATAGTAACTTA
P. che. TAGGTGTAATAATAACAGCAATATTATCAATATATATATATAATAATAGTAATATAATAACAGTATATAATAGTAGCTTA
P. mar. TAGGTATAATAATAACTGTAATAATATCAATATATATATATAATAATAGTAATATAATATCTGTATATAATAGTAACTTA
P. ols. TAGGTATAATAATAACTGTAATAATATCAATATATATATATAATAATAGCAATATAATATCAGTATATAATAGTAACTTA
P. atl. TTATTAATAAATATTAATATATGAATGAAATATAATATATTAGATATGTTAAATTTAAATGTATTAGATTATAAAATATT
P. che. TTATTAATAAATATTAATATATGAATGAAATATAATATATTAGATATGTTAAATTTAAATGTATTAGATTATAAAATATT
P. mar. TTATTAATAAATATTAATATATGAATGAAATATAATATATTAGATATGTTAAATTTAAATGTATTAGATTACAAAATATT
P. ols. TTATTAATAAATATTAATATATGAATGAAATATAATATATTAGATATGTTAAATTTAAATGTATTAGATTATAAAATATT
P. atl. ATTATGGATAGTAGTAATAATTATTAGTAATGAAATATTATTATTTGTAAGTTTCTACTGAATATATTATCATAATTATA
P. che. ATTATGGATAGTAGTAATAATTATTAGTAATGAAATATTATTATTTGTAAGTTTCTACTGAATATATTATCATAATTATA
P. mar. ATTATGGATAGTAGTAATAATTATTAGTAATGAAATATTATTATTTGTAAGTTTCTACTGAATATATTATCATAATTATA
P. ols. ATTATGGATAGTAGTAATAATTATTAGTAATGAAATATTATTATTTGTAAGTTTCTACTGAATATATTATCATAATTATA
P. atl. TAAATTATTATCATGTAAATATATTAATATTAAATTCTTATAACATAGTATATAATTAATATTATATTATTAACATTATTA
P. che. TAAATTATTATCATGTAAATATATTAATATTAAATTCATATAATATAGTATATAACAATATAATATTATTAACATTATTA
P. mar. TAAATTATTATCATGTAAATATATTAATATTAAATTCTTATAATATAGTATATAATTAATATTATATTATTAACATTATTA
P. ols. TAAATTATTATCATGTAAATATATTAATATTAAATTCTTATAACATAGTATATAATTAATATTATATTATTAACATTATTA
P. atl. TCCACAATTGTAAATATAAAATATTATAACTATATATTATCATATTATAAATATATATTAAGTTATTTAATATATTATAA
P. che. TCTACAATTGTAAATATAAAATATTATAATTATATCTTATCATATTATAAATATATATTAAGTTACTTAATATATTATAA
P. mar. TCCACAATTGTAAATATTAAATATTATAATTATATCTTATCATACTATAAATATATATTAAGTTATTTAATATATTATAA
P. ols. TCCACAATTGTAAATATAAAATATTATAACTATATATTATCATATTATAAATATATATTAAGTTATTTAATATATTATAA
P. atl. TAATTATATGTTTAATAATAAAATTATATTAGAATACAAAAATAATAGTAATTATACAAATGATAGTGTATATATGACTA
P. che. TAATTACATGTTTAATAATAAAATTATATTAGAATACAAAAATAATAGTAATTATACAAATGATAGTGTATATATGACTA
P. mar. TAATTATATGTTTAATAATAAAATTATATTAGAATACAAAAATAATAGTAATTATACAAATGATAGTGTATATATGACTA
P. ols. TAATTATATGTTTAATAATAAAATTATATTAGAATACAAAAATAATAGTAATTATACAAATGATAGTGTATATATGACTA
P. atl. GTTATTTCTATATTATATTATTACATTATATACATATATATTATAGTATAATATTAATAGTAGTATATAATTATGTAAAT
P. che. GTTATTTCTATATTATATTATTACATTACATACATATATATTATAGTATCGTATTAATTGTAATATATAATTATGTAAAT
P. mar. GTTATTTCTATATTATATTATTACATTATATTCATATATATTATAGTATAATATTAATAGTAGTATATAATTATGTAAAT
P. ols. GTTATTTCTATATTATATTATTACATTATATACATATATATTATAGTATAATATTAATAGTAGTATATAATTATGTAAAT
P. atl. TATAAAAATATAAATAATTATTATATAAATATAAATATCTATTATCATTTAATAGAAGTATTATATATTATAATATCAAT
P. che. TATAAAAATATAAATAATTATTATATAAATATTAATATCTATTATCATTTAATAGAAGTATTATATATTATTATATCAAT
P. mar. TATAAAAATATAAATAATTATTATATAAATATAAATATCTATTATCATTTAATAGAAGTATTATATATTGTAATATCAAT
P. ols. TATAAAAATATAAATAATTATTATATAAATATAAATATCTATTATCATTTAATAGAAGTATTATATATTATAATATCAAT
P. atl. ATTATTATACTTATAATTATACCATTATTAATAATCTAATATCCTATATTATAGTATA
P. che. ATTATTATACTTATAAATACATATAAATTATAATAATTAATAATTTAATAACGCTAAT
P. mar. ATTATTATACTTATAAATATAAACCAATATATTATTTTACGTGTATAGGTAGTAATAA
P. ols. ATTATTATACTTATAATTATACCATTATTAATAATCTAATATCCTATATTATAGTATA
Conserved 5' Asn-codons
Conserved 5' Phe-codons
Stop codon
